# Utility of the Addenbrooke’s Cognitive Examination III online calculator to differentiate the primary progressive aphasia variants

**DOI:** 10.1093/braincomms/fcac161

**Published:** 2022-07-07

**Authors:** D Foxe, A Hu, S C Cheung, R M Ahmed, N J Cordato, E Devenney, Y T Hwang, G M Halliday, N Mueller, C E Leyton, J R Hodges, J R Burrell, M Irish, O Piguet

**Affiliations:** School of Psychology, The University of Sydney, 94 Mallett St, Sydney, NSW 2006, Australia; Brain and Mind Centre, The University of Sydney, Sydney, NSW 2050, Australia; Brain and Mind Centre, The University of Sydney, Sydney, NSW 2050, Australia; School of Mathematics and Statistics, The University of Sydney, Sydney, NSW 2006, Australia; School of Psychology, The University of Sydney, 94 Mallett St, Sydney, NSW 2006, Australia; Brain and Mind Centre, The University of Sydney, Sydney, NSW 2050, Australia; Brain and Mind Centre, The University of Sydney, Sydney, NSW 2050, Australia; Central Clinical School, The University of Sydney, Sydney, NSW 2006, Australia; Brain and Mind Centre, The University of Sydney, Sydney, NSW 2050, Australia; St George Clinical School, University of New South Wales, Sydney, NSW 2217, Australia; The Department of Aged Care, St George Hospital, Sydney, NSW 2217, Australia; Calvary Health Care Kogarah, Calvary Community Health, Sydney, NSW 2217, Australia; Brain and Mind Centre, The University of Sydney, Sydney, NSW 2050, Australia; Central Clinical School, The University of Sydney, Sydney, NSW 2006, Australia; Brain and Mind Centre, The University of Sydney, Sydney, NSW 2050, Australia; Central Clinical School, The University of Sydney, Sydney, NSW 2006, Australia; Brain and Mind Centre, The University of Sydney, Sydney, NSW 2050, Australia; Central Clinical School, The University of Sydney, Sydney, NSW 2006, Australia; Brain and Mind Centre, The University of Sydney, Sydney, NSW 2050, Australia; Central Clinical School, The University of Sydney, Sydney, NSW 2006, Australia; School of Psychology, The University of Sydney, 94 Mallett St, Sydney, NSW 2006, Australia; Brain and Mind Centre, The University of Sydney, Sydney, NSW 2050, Australia; Brain and Mind Centre, The University of Sydney, Sydney, NSW 2050, Australia; Brain and Mind Centre, The University of Sydney, Sydney, NSW 2050, Australia; Concord Clinical School, Sydney Medical School, The University of Sydney, Sydney, NSW 2139, Australia; School of Psychology, The University of Sydney, 94 Mallett St, Sydney, NSW 2006, Australia; Brain and Mind Centre, The University of Sydney, Sydney, NSW 2050, Australia; School of Psychology, The University of Sydney, 94 Mallett St, Sydney, NSW 2006, Australia; Brain and Mind Centre, The University of Sydney, Sydney, NSW 2050, Australia

**Keywords:** frontotemporal dementia, Alzheimer’s disease, cognitive assessment, dementia screening, data-driven classification

## Abstract

The Addenbrooke’s Cognitive Examination III is a brief cognitive screening tool that is widely used for the detection and monitoring of dementia. Recent findings suggest that the three variants of primary progressive aphasia can be distinguished based on their distinct profiles on the five subdomain scores of this test. Here, we investigated the utility of the Addenbrooke’s Cognitive Examination III to differentiate the primary progressive aphasia variants based on their item-by-item performance profiles on this test. From these results, we created an interactive primary progressive aphasia Addenbrooke’s Cognitive Examination III calculator which predicts the variant based on a patient’s unique item-by-item profile. Twenty-eight logopenic variant, 25 non-fluent variant and 37 semantic variant primary progressive aphasia patients and 104 healthy controls completed the Addenbrooke’s Cognitive Examination III at first clinical presentation. Multinomial regression analyses were conducted to establish performance profiles among groups, and R Shiny from RStudio was used to create the interactive Addenbrooke’s Cognitive Examination III diagnostic calculator. To verify its accuracy, probability values of the regression model were derived based on a 5-fold cross-validation of cases. The calculator’s accuracy was then verified in an independent sample of 17 logopenic, 19 non-fluent and 13 semantic variant primary progressive aphasia patients and 68 Alzheimer’s disease patients who had completed the Addenbrooke’s Cognitive Examination III (or an older version of this test: Revised) and had *in vivo* amyloid-PET imaging and/or brain autopsy pathological confirmation. Cross-validation of cases in the calculator model revealed different rates of sensitivity in classifying variants: semantic = 100%, non-fluent = 80.6% and logopenic = 79.9%; healthy controls were distinguished from primary progressive aphasia patients with 100% sensitivity. Verification of *in vivo* amyloid and/or autopsy-confirmed patients showed that the calculator correctly classified 10/13 (77%) semantic variant, 3/19 (16%) non-fluent variant and 4/17 (24%) logopenic variant patients. Importantly, for patients who were not classified, diagnostic probability values mostly pointed toward the correct clinical diagnosis. Furthermore, misclassified diagnoses of the primary progressive aphasia cohort were rare (1/49; 2%). Although 22 of the 68 Alzheimer’s disease patients (32%) were misclassified with primary progressive aphasia, 19/22 were misclassified with the logopenic variant (i.e. falling within the same neuropathological entity). The Addenbrooke’s Cognitive Examination III primary progressive aphasia diagnostic calculator demonstrates sound accuracy in differentiating the variants based on an item-by-item Addenbrooke’s Cognitive Examination III profile. This calculator represents a new frontier in using data-driven approaches to differentiate the primary progressive aphasia variants.

## Teaser

This study promotes the Addenbrooke’s Cognitive Examination III primary progressive aphasia diagnostic calculator. This interactive tool demonstrates sound accuracy in differentiating the primary progressive aphasia variants based on an item-by-item Addenbrooke’s Cognitive Examination III profile. It is freely available for use in clinical settings. http://shiny.maths.usyd.edu.au/PPA_diagnostic_calc/

## Graphical Abstract

**Graphical abstract:**
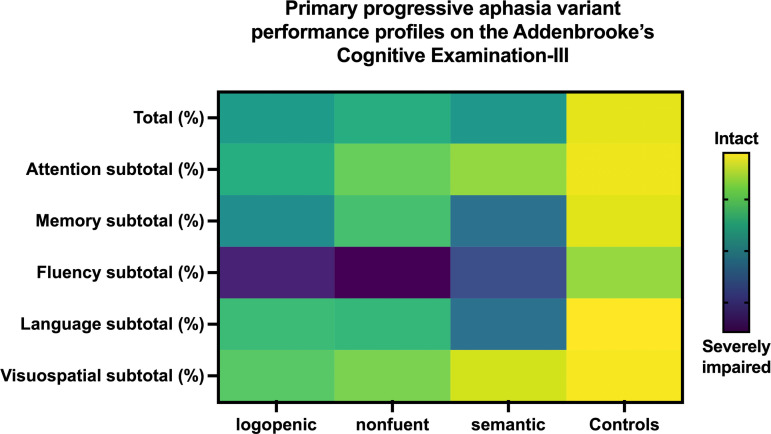

## Introduction

Diagnosing younger-onset dementia in clinical settings remains complex and challenging.^1^ The clinical features often overlap and are difficult to distinguish from other disorders of aging and mood, which can lead to delayed diagnosis and subsequent access to appropriate support services.^1,2^ This is particularly true for primary progressive aphasia (PPA), a group of younger-onset dementias characterized clinically by early and marked disturbances in language abilities.^3–5^ Three PPA variants are recognised based on specific profiles of language, patterns of brain atrophy, and neuropathology: (i) logopenic variant-PPA (lv-PPA), (ii) non-fluent variant-PPA (nfv-PPA) and (iii) semantic variant-PPA (sv-PPA).^3^ Differentiating the PPA variants is important given the distinct prognoses, functional needs and underlying neuropathological processes—in brief, sv-PPA and nfv-PPA are predominantly associated with frontotemporal lobar degeneration (FTLD) pathology (TDP-43 and Tau, respectively), whereas lv-PPA is primarily associated with Alzheimer’s disease pathology.^6–9^ Accurate classification of these variants therefore is crucial to improve clinical care and ensure patients are triaged to the most relevant clinical interventions and support services.^10–12^

Despite their respective diagnostic criteria, classifying the PPA variants is challenging as the language features often overlap or do not fit neatly into the subtyping scheme.^4,5,13^ A growing body of research suggests that measures of cognition (beyond language ability) may improve the diagnostic accuracy of PPA due to the distinct nature of evolving cognitive deficits among the variants.^14–16^ For example, recent findings indicate that lv-PPA progresses more rapidly to a diffuse/global dementia syndrome relative to nfv-PPA and sv-PPA,^17–20^ with the suggestion that notable declines on measures of orientation, attention, visuospatial memory, and visuospatial abilities differentiate lv-PPA from the other PPA variants.^14,15,17,20–24^ These findings are especially relevant in differentiating between lv-PPA and nfv-PPA as the language features of these syndromes commonly intersect or appear nuanced to inexperienced clinicians.^4,13,25^

Importantly, recent research suggests the PPA cognitive profiles are distinguishable using a brief cognitive screening instrument—the Addenbrooke’s Cognitive Examination III (ACE-III).^26^ The ACE-III is widely used to detect and monitor dementia and examines the integrity of five cognitive domains [Attention and Orientation (/18), Memory (/26), Verbal Fluency (/14), Language (/26), and Visuospatial (/16)], summed to create an overall cognition score (/100).^26–28^ Existing evidence points to the utility of the ACE-III and its predecessor, the ACE-Revised (ACE-R), in detecting the early stages of PPA and classifying the PPA variants based on their distinct profiles across the cognitive domains.^17,18,20,23,29,30^ Moreover, longitudinal studies indicate that declines on the ACE-III Attention and Visuospatial subdomains are more pervasive in lv-PPA than in the other PPA variants, and, when Attention and Visuospatial ability remain relatively preserved, early impairment on the Language and Memory subdomains is more characteristic of sv-PPA than nfv-PPA.^17,20^ Put together, these findings demonstrate the utility of the ACE-III in characterizing the PPA variants based on distinct cognitive profiles throughout the disease course.

Investigations in other dementia populations demonstrate that the efficacy of general screening measures to differentiate dementia subtypes is improved when considering the pattern of individual item scores rather than the overall, summed score.^31,32^ Building on these findings, the question arises as to whether the ACE-III would demonstrate improved accuracy in discriminating the PPA variants by considering its test items and their interactions. Conducting such item analyses, however, involves complex statistical methods and requires the interpretation of many permutations of outcomes, somewhat defeating the purpose of having a brief and simple clinical tool. With the recent advancement of technology, however, interactive statistical tools are now available which, built on modelling data, can interpret the unique item-by-item performance of an individual patient and provide key clinical information for diagnosis and management.^32^

The aims of this study were to (i) determine the ACE-III items that differentiate the PPA variants, (ii) use multinomial regression modelling to create an online PPA diagnostic calculator based on the pattern of performance on the ACE-III and (iii) verify the reliability of the ACE-III PPA diagnostic calculator in a neuropathologically confirmed sample of PPA patients.

## Materials and methods

### Participants

#### Phase 1: evaluating the psychometric properties of the Addenbrooke’s Cognitive Examination III in a verified primary progressive aphasia cohort

**Figure 1:**
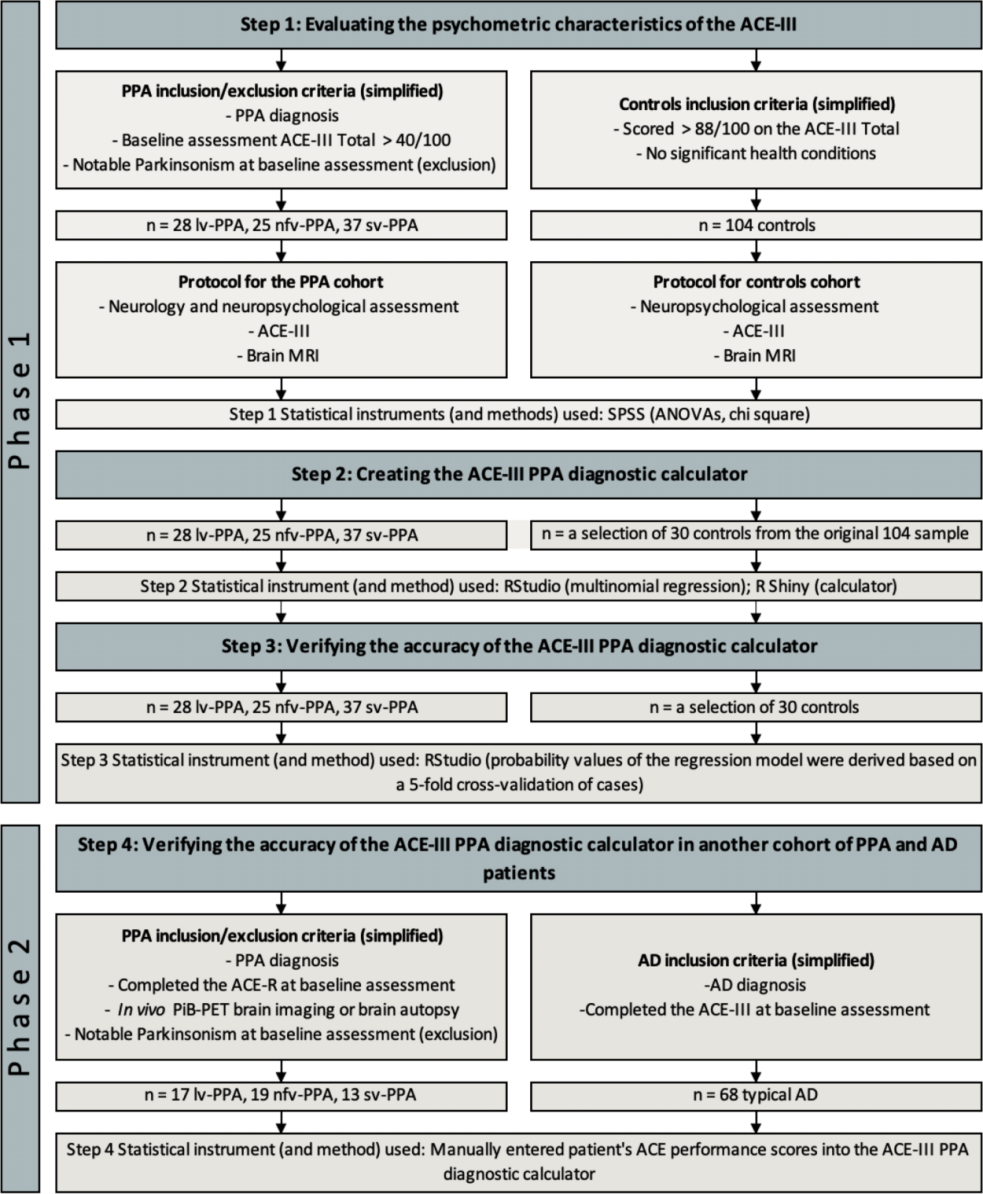
**Flow chart of the study design.** ACE-III = Addenbrooke’s Cognitive Examination-Third Edition; ACE-R = Addenbrooke’s Cognitive Examination-Revised; ANOVA = analysis of variance; Controls = healthy control volunteers; DAD = Disability Assessment for Dementia; lv-PPA = logopenic variant primary progressive aphasia; MRI = magnetic resonance imaging; nfv-PPA = non-fluent variant primary progressive aphasia; PiB-PET = [11C] Pittsburgh compound B positron emission tomography; SPSS = IBM Statistical Package for the Social Sciences; sv-PPA = semantic variant primary progressive aphasia

To evaluate the psychometric properties of the ACE-III and create a model (*Aims 1 and 2*), 90 patients diagnosed with PPA (28 lv-PPA, 25 nfv-PPA, 37 sv-PPA) and 104 healthy matched controls were recruited from FRONTIER, a younger-onset dementia research clinic based in Sydney, Australia, between October 2010 and February 2020 (Fig. 1). Patients underwent comprehensive clinical assessment with an experienced neurologist, neuropsychological assessment, and a structural brain MRI scan. Diagnosis was considered at each clinical visit and was established according to the relevant clinical diagnostic criteria at the time of testing.^3^ Their baseline assessment data were used for this study. Patients were excluded if they scored below 40/100 on the ACE-III^26^ at first visit, were not proficient in English, or did not have a reliable informant. Patients were also excluded if they had a clear or suspected parkinsonian syndrome (see Supplementary Methods and Supplementary Fig. 1), had a history of psychiatric illness, brain injury or substance abuse.

Control participants were selected from a healthy volunteer panel and were included in the study if they scored > 88/100 on the ACE-III^26,33^ and did not have a history of psychiatric illness, brain injury, family history of dementia, or substance abuse. Controls were required to have a reliable informant to complete survey material [i.e. questionnaires about their cognitive abilities and activities of daily living (ADLs)]. A subset of controls was randomly selected from the original sample to create the ACE-III PPA diagnostic calculator (*Aim 2*) (Fig. 1).

#### Phase 2: verification of the integrity of the online Addenbrooke’s Cognitive Examination III primary progressive aphasia diagnostic calculator

To confirm the accuracy of the online ACE-III PPA diagnostic calculator (*Aim 3*), 49 patients diagnosed with PPA (13 typical and 4 atypical lv-PPA, 12 typical and 7 atypical nfv-PPA, 9 typical and 4 atypical sv-PPA) not included in the initial evaluation of the ACE-III and calculator data modelling were selected, in addition to 68 patients diagnosed with typical Alzheimer’s disease (Fig. 1). These patients were also recruited from FRONTIER (PPA patients between November 2007 and August 2013; Alzheimer’s disease patients between October 2008 and February 2020) and underwent the same assessment procedure as the cohort in Phase 1. These PPA patients were selected based on the availability of at least one of following: (i) [11C] Pittsburgh compound B PET (PiB-PET) findings (occurring within the first 3 years of their baseline assessment at FRONTIER); (ii) baseline evaluation using the Progressive Aphasia Language Scale (PALS)^34^ (Supplementary Table 1 and Appendix A1); and/or, (iii) post-mortem brain pathology findings. As the PPA patients were longstanding FRONTIER participants, their clinical assessments predate the creation of the ACE-III. Their baseline ACE-R scores were thus converted to equivalent ACE-III scores for this study (Supplementary Table 7). The Alzheimer’s disease patients were selected based on availability of ACE-III (not ACE-R; no conversion required) scores and, where available, PiB-PET findings. The majority of Alzheimer’s disease patients completed the ACE-III at baseline assessment; for a small number of patients (*n* = 10), ACE-III baseline results were not available, and ACE-III results from their second annual visit were used instead. PPA and Alzheimer’s disease patients were excluded if they scored below 40/100 on either the ACE-R or ACE-III.

### Ethics statement

All participants or their person responsible (i.e. informant, spouse, and so on) provided written informed consent in accordance with the Declaration of Helsinki. This study was approved by the Human Research Ethics Committee of the South Eastern Sydney Local Area Health District (HREC 10/126) and the University of New South Wales Ethics Advisory panel D (Biomedical, ref. # 10035).

### Clinical measures

#### General cognition and functional capacity

In addition to completing the ACE-III^26^ (Phase 1: all PPA patients, controls; Phase 2: Alzheimer’s disease patients) or ACE-R^35^ (Phase 2: all PPA patients), participants underwent a comprehensive neuropsychological assessment, details of which are provided in in the Supplementary material.

The Disability Assessment for Dementia (DAD) was also administered through interview by an experienced research occupational therapist or psychologist to the patients’ spouses, relatives or carers.^36^ The DAD is an informant-based measure which assesses the patient’s functional capacity in completing ADLs. The total DAD score is reported as a percentage, with lower scores reflecting greater functional impairment. The controls’ informant completed a questionnaire version of this assessment.

#### Additional measures: [11C] Pittsburgh compound B-PET imaging, Progressive Aphasia Language Scale, neuropathology

PiB-PET was used to select PPA patients for the purposes of confirming the accuracy of the ACE-III PPA diagnostic calculator (Phase 2); the majority of PPA patients (47/49; 96%) and a minority of Alzheimer’s disease patients (5/68; 7%) underwent PiB-PET. PiB-PET uses a radio-ligand of amyloid protein as a biomarker for Alzheimer’s disease.^37^ Consistent with prior studies,^34^ high PiB cortical uptake (i.e. ‘Positive’ PiB-PET) was defined as a standardized cortical uptake value ratio (SUVR), with the cerebellar cortex as a reference region, equal or higher than 1.5. ‘Equivocal’ PiB-PET was defined as a SUVR between 1.2 and 1.4^38^ and a ‘Negative’ PiB-PET was defined as a SUVR binding below 1.2.

Most PPA patients in Phase 2 completed the PALS (38/49; 78%).^34^ The PALS measures the magnitude and consistency of impairments in seven features of language ability: motor speech, agrammatism, naming, single-word repetition, single-word comprehension, sentence repetition, and sentence comprehension (Supplementary Table 1). Each feature is scored as absent (0), subtle or questionable (1), mild but definitely present (2), or moderate/severe (3) (see instructions of the PALS in Supplementary Material Appendix A1).

Neuropathological diagnosis was confirmed in a subset of PPA patients in Phase 2 (17/49; 35%). Methods for neuropathological diagnosis have been previously described.^39^ In brief, routine neuropathologic evaluation, based on established immunohistochemical inclusions,^40,41^ was conducted to determine the pathologic subtype, namely, Alzheimer’s disease, FTLD with tau inclusions, and FTLD with transactive response DNA-binding protein 43 (TDP-43) inclusions in any of their subtypes TDP-43 type A, B, C, or D.^40^

### Statistical analysis

Data were analysed using IBM SPSS Statistics, 24.0 (SPSS Inc., Chicago, Ill., USA) and RStudio v1.2.5042 (RStudio Team 2020, Boston, MA, USA). Figures were created with RStudio and GraphPad Prism 8 (GraphPad Software).

#### Evaluation of demographic, Addenbrooke’s Cognitive Examination III and Disability Assessment for Dementia characteristics (Phase 1, Step 1)

The distribution normality of demographic, cognitive and behavioural data were first determined with Shapiro–Wilks tests. Variables with normal distributions were compared across groups using one-way analyses of variance (ANOVAs) followed by Sidak post hoc tests. Variables not normally distributed were analysed using Kruskal–Wallis tests followed by pairwise comparisons, Bonferroni corrected. χ^2^ were used to analyse categorical measures (e.g. sex) followed by *z*-tests to compare column proportions, Bonferroni corrected.

#### Creation and evaluation of the Addenbrooke’s Cognitive Examination III primary progressive aphasia diagnostic calculator (Phase 1 Step 2 and Phase 1 Step 3)

R Shiny from RStudio was used to create the interactive ACE-III PPA diagnostic calculator. To build the underlying calculator, 30 control participants were randomly sampled to minimise class imbalance for model training (Fig. 1). A penalised multinomial logistic regression classifier with a ridge penalty was fitted to perform diagnostic predictions. Since many data points (i.e. ACE-III test scores within a cognitive subdomain) were multicollinear (i.e. where independent variables are highly correlated), a ridge penalty was imposed on the regression coefficients to perform L2 regularization. The amount of penalty was fine-tuned using a constant lambda (λ); adjusting the value of lambda by testing different lambda values allowed for model tuning to find the best ridge penalised multinomial regression model. The optimal λ value (λ = 0.044) was identified using cross-validation, where the loss function used to fit the regression was determined by the misclassification error (misclassification error = 0.18, SE = 0.032). Classification thresholds (*α*) were set to minimize misclassification error: *α*_*lv*−*PPA*_  = 0.7, *α*_*nfv*−*PPA*_  = 0.7, *α*_*sv*−*PPA*_  = 0.75, and *α*_*control*_  = 0.7. That is, if the predicted probabilities were above these thresholds, a prediction was given. Predicted probabilities below these values were classified as ‘uncertain’. The regression model was also used to conduct analyses to establish the ACE-III performance profiles among PPA variants using the model coefficients and *P*-values.

One hundred repetitions of 5-fold cross-validation were applied to evaluate the model performance (Phase 1 Step 3). Then, sensitivity (true positive rate) and specificity (true negative rate) evaluation metrics were inspected to determine the model’s (i.e. ACE-III PPA calculator’s) accuracy. In this study, sensitivity refers to the probability of a case (i.e. individual) within the model being correctly labelled with their true condition (i.e. the diagnosis provided by the neurologist) (positive test). In contrast, specificity refers to the probability of a case (i.e. individual) within the model being correctly labelled as not having the condition (a true negative test; conditioned on truly being negative). All patients and controls were included when evaluating the sensitivity and specificity valuations for each diagnostic group. Four separate analyses were conducted (i.e. lv-PPA, nfv-PPA, sv-PPA and controls), with the ‘positive’ versus ‘negative’ labelling varying per analysis (e.g. for testing lv-PPA, sensitivity rates were concerned with ‘lv-PPA’ versus ‘not lv-PPA’).

### Data availability

The ethical requirement to ensure patient confidentiality precludes public archiving of our data. Researchers who would like to access the raw data should contact the corresponding author, who will liaise with the ethics committee that approved the study, and accordingly, as much data that are required to reproduce the results will be released to the individual researcher. The codes used for this project are available for review on the Open Science Framework website (https://osf.io/7rwk6/). The ACE-III is freely available at frontierftd.org and the online ACE-III PPA diagnostic calculator created for this study is freely available at http://shiny.maths.usyd.edu.au/PPA_diagnostic_calc/. In Supplementary Table 2, we have provided an appendix which clarifies the ACE-III item terms/descriptors used in this study and the terms/descriptors used in the online calculator. Legal copyright restrictions prevent public archiving of the other neuropsychological tests used in this research. These materials can be obtained from the copyright holders in the cited references. No part of the study procedures or analyses were preregistered prior to the research being undertaken.

## Results

### Demographics (Phase 1 Step 1)

Participants did not differ significantly for sex or age, but differed in terms of education, with lv-PPA patients completing fewer years of education than controls (mean difference = 1.9 years; *P* = 0.022) (Table 1). Patients with PPA were comparable for disease duration (i.e. years since symptom onset) and DAD scores. Significant group differences were observed for the ACE-III total score, where all patient groups scored significantly lower than controls (all *P-*values < 0.001) and the sv-PPA group scored significantly lower than the nfv-PPA group (*P* = 0.039). Group differences on the ACE-III subdomains are displayed in Table 1 and reported in Supplementary material. The demographic characteristics of the 30 controls used in Phase 1 Step 2 are displayed in Supplementary Table 3.

**Table 1:** Demographic variables of primary progressive aphasia patients and healthy controls

	lv-PPA	nfv-PPA	sv-PPA	Controls	F	*P*	*Post hoc* test (Sidak corrected)
Sex (m: f)	15:13	9:16	18:19	49:55	1.740†	0.628	
Age (y)	66.1 (6.6)	67.2 (10.2)	64.3 (7.2)	67 (7.8)	1.168	0.323	
Education (y)	12.2 (3)	12.5 (2.7)	13 (2.9)	14.1 (2.9)	4.297	0.006	lv-PPA < Controls
Disease duration (y)	3.5 (2.3)	4.9 (3)	4.7 (2.4)	N/A	2.450	0.092	
DAD Total (/100)	80.7 (18.3)	78.9 (19.1)	81.4 (16.3)	N/A	0.147	0.863	
ACE-III Total (/100)	66.4 (15.1)	71.8 (13.1)	65.3 (11.8)	95.2 (2.9)	147.958	<0.001	sv-PPA < nfv-PPA; PPA < Controls
ACE-III Attention (/18)	12.9 (3.5)	14.8 (2.7)	15.6 (1.7)	17.3 (1)	45.049	<0.001	lv-PPA < nfv-PPA, sv-PPA < Controls
ACE-III Memory (/26)	16.2 (5.6)	20.2 (5.9)	14.2 (4.8)	24.7 (1.4)	88.127	<0.001	lv-PPA, sv-PPA < nfv-PPA; PPAs < Controls
ACE-III Fluency (/14)	4.9 (2.8)	4 (2.4)	6.3 (3)	12.2 (1.5)	169.899	<0.001	nfv-PPA < sv-PPA; PPAs < Controls
ACE-III Language (/26)	19.7 (5)	19.4 (3.5)	14.2 (4.5)	25.5 (0.8)	136.932	<0.001	sv-PPA < lv-PPA, nfv-PPA < Controls
ACE-III Visuospatial (/16)	12.8 (2.8)	13.5 (2.2)	14.9 (1.2)	15.6 (0.7)	31.452	<0.001	lv-PPA, nfv-PPA < sv-PPA, Controls

Notes: Values are mean ± standard deviation. † = χ^2^ test.

ACE-III = Addenbrooke’s Cognitive Examination-Third edition; Education = total years formal education; DAD Total = Disability Assessment for Dementia total score; Disease duration = time (years) since the onset of symptoms as described by the caregiver; (f) =  female; lv-PPA = logopenic variant primary progressive aphasia; (m) =  male; nfv-PPA = non-fluent variant primary progressive aphasia; sv-PPA = semantic variant primary progressive aphasia; (y) = years.

### ACE-III items (Phase 1 Step 1)

**Figure 2:**
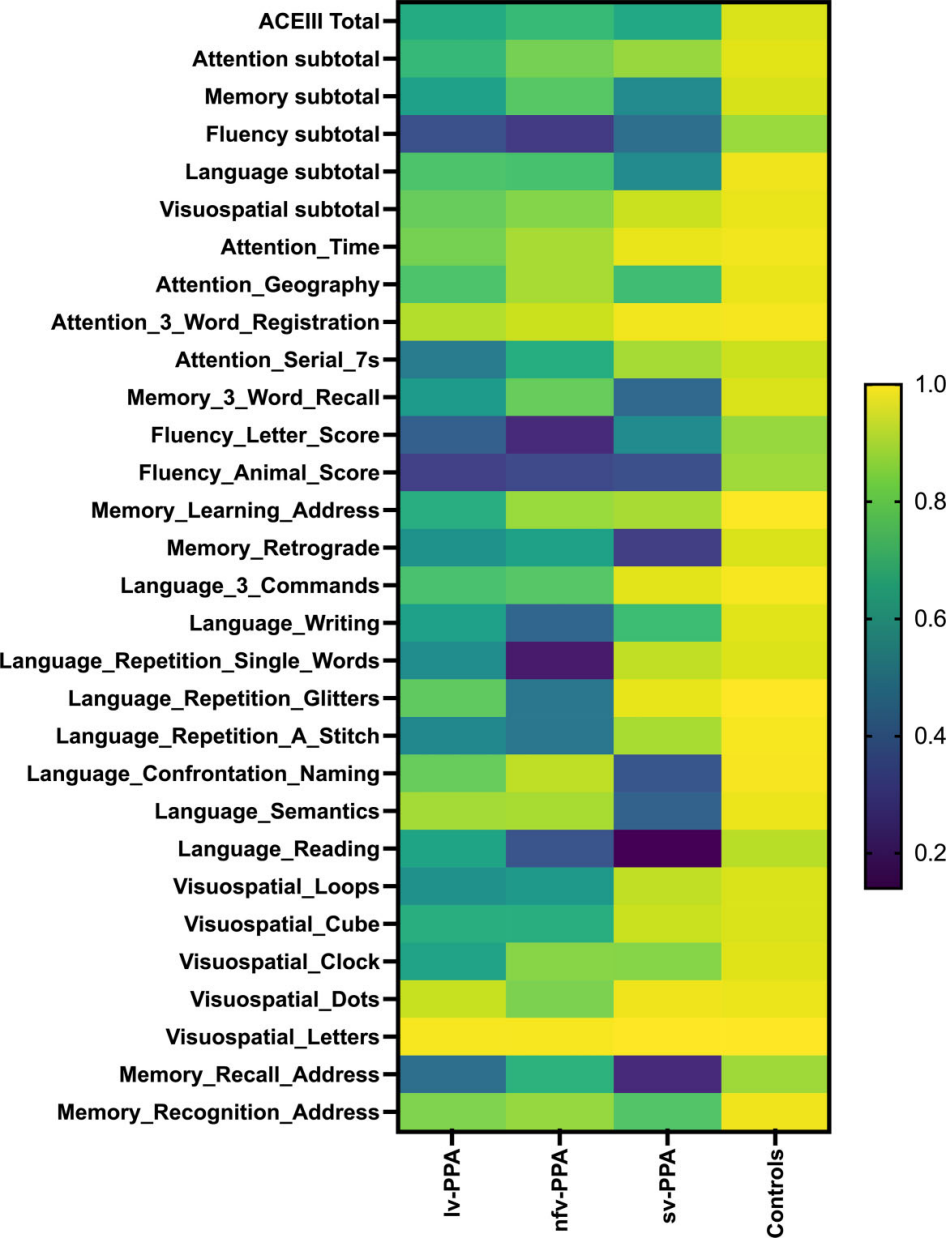
**Heat map of the ACE-III Total, subdomain, and item scores in 90 primary progressive aphasia patients and 104 controls.** Group mean scores are represented as a percentage of the total score (i.e. mean/total score ×100). Darker colours reflect poorer performance. ACE-III labels used in this figure and in the online PPA ACE-III diagnostic calculator are clarified in Supplementary Table 2

**Figure 3:**
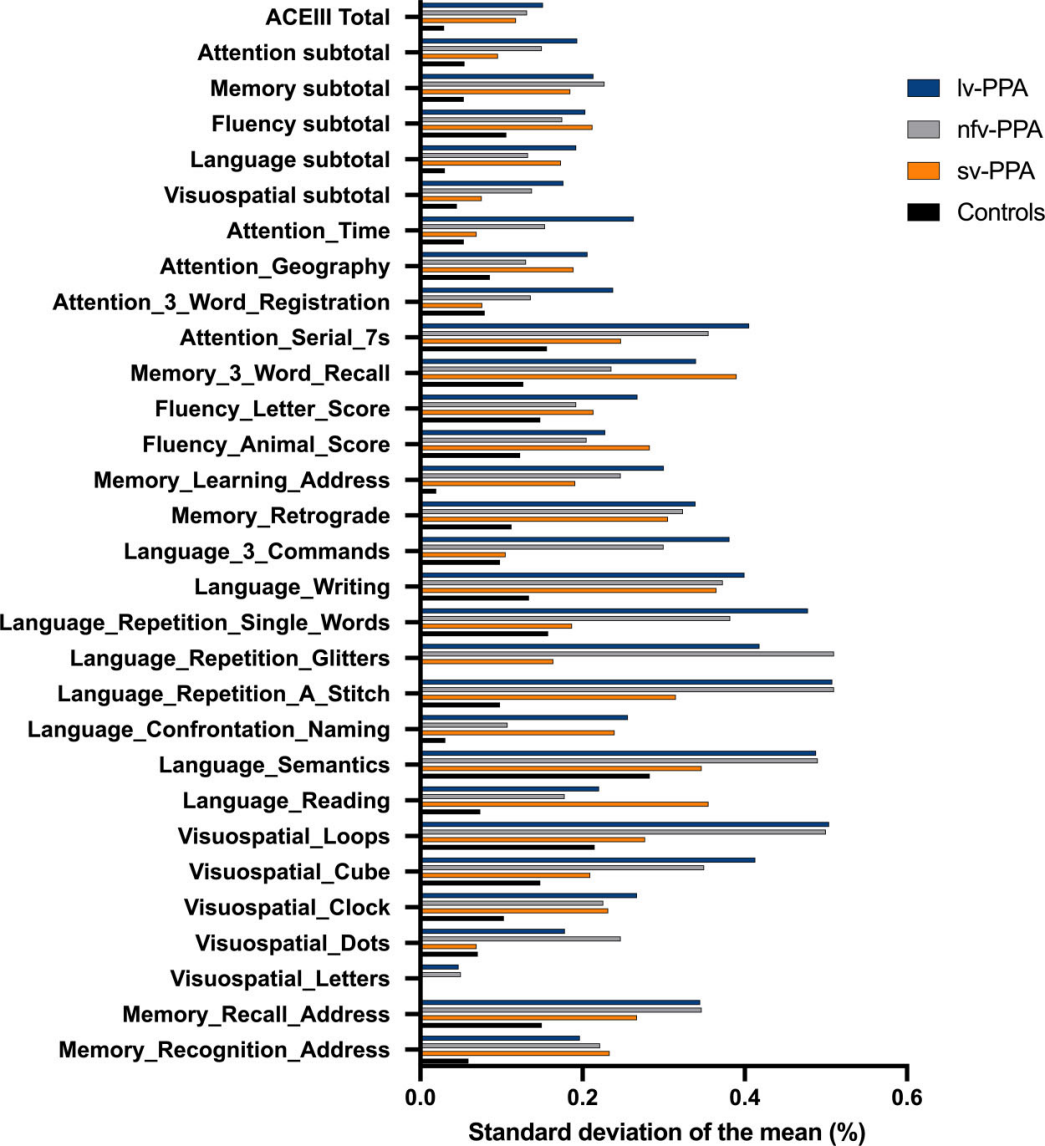
**Performance variability among primary progressive aphasia variants (*n* = 90) and controls (*n* = 104) on the ACE-III Total, cognitive subdomains and test items.** Bars represent the group standard deviations of the group mean. ACE-III labels used in this figure and in the online PPA ACE-III diagnostic calculator are clarified in Supplementary Table 2

Group means of the ACE-III total, subdomain and item scores are represented as percentages of the total score (i.e. group mean/total score × 100) and displayed as a heat map (Fig. 2). Standard deviations of the group means of these measures are displayed as bar charts in Fig. 3. A detailed description of the ACE-III item profiles is provided in the Supplementary material and ACE-III item raw scores and *post hoc* group comparisons are presented in Supplementary Table 5. Performance on ACE-III items within each PPA group was characteristic of their respective clinical variant profile. Relative to controls, the lv-PPA group demonstrated widespread cognitive difficulties, predominantly in verbal learning and memory, verbal fluency, and aspects of attention and visuoconstruction (Supplementary Table 4). By contrast, the nfv-PPA group displayed a more circumscribed profile with impairments in verbal output and aspects of attention and visuoperception. Verbal learning and memory, however, were relatively preserved. Finally, the sv-PPA group demonstrated the most circumscribed profile, with predominant deficits on items involving conceptual knowledge either directly (i.e. naming, object comprehension) or indirectly (i.e. verbal fluency, verbal memory). Conversely, verbal repetition, visuospatial abilities, and most aspects of attention remained relatively preserved. Importantly, ACE-III performance profiles across and within PPA groups were more salient at an item-by-item level than at the subdomain level (i.e. Attention, Memory, Language, and so on) (Fig. 2). In addition, and unsurprisingly, variability in performance (i.e. standard deviation from the mean) was more pronounced at an item-by-item level than at the subdomain level, especially on items with a small scoring range (i.e. 0–1) (Fig. 3).

#### Model evaluation using cross-validation (Phase 1 Step 3)

The multinomial deviance for the ridge penalised regression model resulted in R^2^ = 0.833, *d* = 24; that is, 83.3% of the variance for the diagnosis was explained by the ACE-III test scores. After 100 repetitions of 5-fold cross-validation, we recorded the overall model accuracy, the sensitivity, specificity, and precision rates (i.e. if the predicted probabilities surpassed the classification threshold, a ‘more certain’ prediction was given). The overall model accuracy (i.e. if a prediction is given) was 94.3%. The PPA sensitivity rates [i.e. the ability of the model to correctly classify cases of interest (e.g. lv-PPA) from all the other cases (i.e. nfv-PPA, sv-PPA, controls)] were as follows: lv-PPA = 79.9%, nfv-PPA = 80.6%, and sv-PPA = 100%; controls were distinguished from PPA cases with a sensitivity rate of 100%. The specificity rates were lv-PPA = 96.6%, nfv-PPA = 96.9%, and sv-PPA = 99.8%, and controls = 100%. The precision rates were lv-PPA = 81.3%, nfv-PPA = 84.3%, sv-PPA = 99.6%, and controls = 100%. From all test cases conducted in the 5-fold cross-validation, the proportion of ‘uncertain’ values was 36.7%; in other words, the model yielded a prediction for almost two-thirds (63.3%) of the test cases. Of the test cases that gave a prediction, the PPA variant proportions were lv-PPA = 24.7%, nfv-PPA = 47.9%, sv-PPA = 71%, and controls = 88.9% (e.g. of all the test cases where lv-PPA was the clinical diagnosis, 24.7% gave a prediction while 75.3% were N/A values; for all the test cases where nfv-PPA was the actual diagnosis, 47.9% gave a prediction while 53.1% were N/A values, and so on).

#### Confirmation of the Addenbrooke’s Cognitive Examination III primary progressive aphasia diagnostic calculator in [11C] Pittsburgh compound B-PET and/or autopsy-confirmed primary progressive aphasia patients (Phase 2 Step 4)

Verification of *in vivo* amyloid confirmed and/or autopsy PPA patients revealed correct classification of 10/13 (77%) sv-PPA, 3/19 (16%) nfv-PPA, and 4/17 (24%) lv-PPA patients (Table 2). Importantly, for patients who did not reach a statistical threshold for diagnostic classification, probability values mostly pointed toward the correct clinical diagnosis. Further, misclassified diagnoses were extremely rare (1/49; 2%), with only one lv-PPA patient misclassified as sv-PPA (Table 2).

**Table 2:** Application of the Addenbrooke’s Cognitive Examination III primary progressive aphasia diagnostic calculator in PiB-PET or autopsy-confirmed patients

Patient (Dx according to neurologist)	Dx certainty	Age at PALS and/or ACE-R	PALS diagnosis	ACE-R Total score	PiB-PET result	Brain pathology	ACE-III PPA diagnostic calculator				
							lv-PPA versus nfv-PPA calculator prediction	sv-PPA versus non sv-PPA calculator prediction	lv-PPA probability	nfv-PPA probability	sv-PPA probability
lv-PPA case 1	Certain	68	lv-PPA	72	Positive	Alzheimer’s disease *	Uncertain	Not sv-PPA	0.46	0.22	0.24
lv-PPA case 2	Certain	73	Unclassifiable	69	Positive		lv-PPA	Not sv-PPA	0.73	0.12	0.10
lv-PPA case 3	Certain	56	lv-PPA	58	Positive		Uncertain	Not sv-PPA	0.70	0.08	0.21
lv-PPA case 4	Certain	72	lv-PPA	74	Positive		Uncertain	Not sv-PPA	0.36	0.13	0.45
lv-PPA case 5	Certain	59	lv-PPA	63	Positive		Uncertain	Not sv-PPA	0.39	0.11	0.48
lv-PPA case 6	Certain	61	nfv-PPA	58	Positive	Alzheimer’s disease	Uncertain	Not sv-PPA	0.52	0.41	0.05
lv-PPA case 7	Certain	69	lv-PPA	55	Positive		lv-PPA	Not sv-PPA	0.87	0.07	0.05
lv-PPA case 8	Certain	77	Unclassifiable	90	Positive		Uncertain	Not sv-PPA	0.23	0.40	0.04
lv-PPA case 9	Certain	63	Unclassifiable	56	Positive		Uncertain	Not sv-PPA	0.32	0.30	0.36
lv-PPA case 10	Certain	75	Not available	44	N/A	Alzheimer’s disease *	Uncertain	Not sv-PPA	0.63	0.23	0.15
lv-PPA case 11	Certain	53	lv-PPA	68	Positive		Uncertain	Not sv-PPA	0.36	0.25	0.38
lv-PPA case 12	Certain	59	lv-PPA	63	Positive		Uncertain	Not sv-PPA	0.36	0.22	0.39
lv-PPA case 13	Certain	70	nfv-PPA	74	Positive	FTLD-Tau^1^	lv-PPA	Not sv-PPA	0.73	0.04	0.13

lv-PPA case 14	Uncertain	65	lv-PPA	44	Positive		lv-PPA	Not sv-PPA	0.94	0.04	0.03
lv-PPA case 15	Uncertain	71	Unclassifiable	64	Positive		Uncertain	Not sv-PPA	0.40	0.19	0.39
lv-PPA case 16	Uncertain	65	lv-PPA	60	Equivocal	Alzheimer’s disease	Not lv-PPA; Not nfv-PPA	sv-PPA	0.11	0.02	0.86
lv-PPA case 17	Uncertain	65	Unclassifiable	75	Negative		Uncertain	Not sv-PPA	0.60	0.14	0.17

nfv-PPA case 1	Certain	55	nfv-PPA	62	Negative		Uncertain	Not sv-PPA	0.31	0.48	0.19
nfv-PPA case 2	Certain	80	Not available	41	N/A	FTLD-Tau^2^	Uncertain	Not sv-PPA	0.54	0.43	0.00
nfv-PPA case 3	Certain	78	nfv-PPA	56	Equivocal	FTLD-Tau^3^*	Uncertain	Not sv-PPA	0.35	0.63	0.01
nfv-PPA case 4	Certain	71	Unclassifiable	83	Negative	FTLD-Tau^3^	Uncertain	Not sv-PPA	0.20	0.39	0.20
nfv-PPA case 5	Certain	47	nfv-PPA	87	Negative	FTLD-Tau^1^*	Uncertain	Not sv-PPA	0.24	0.53	0.05
nfv-PPA case 6	Certain	70	nfv-PPA	89	Negative		Uncertain	Not sv-PPA	0.21	0.52	0.03
nfv-PPA case 7	Certain	72	Not available	75	Negative	FTLD-Tau^1^*	nfv-PPA	Not sv-PPA	0.08	0.86	0.03
nfv-PPA case 8	Certain	77	Not available	74	Negative		nfv-PPA	Not sv-PPA	0.13	0.74	0.11
nfv-PPA case 9	Certain	73	Not available	87	Negative		Uncertain	Not sv-PPA	0.36	0.26	0.19
nfv-PPA case 10	Certain	60	Not available	85	Negative	FTLD-Tau	Uncertain	Not sv-PPA	0.14	0.63	0.11
nfv-PPA case 11	Certain	70	nfv-PPA	85	Negative	FTLD-Tau^4^	Uncertain	Not sv-PPA	0.34	0.40	0.04
nfv-PPA case 12^	Certain	63	Not available	74	Negative	FTLD-Tau	Uncertain	Not sv-PPA	0.39	0.52	0.05

nfv-PPA case 13	Uncertain	71	Not available	45	Equivocal		Uncertain	Not sv-PPA	0.68	0.30	0.02
nfv-PPA case 14	Uncertain	70	Not available	71	Negative		Uncertain	Not sv-PPA	0.30	0.57	0.11
nfv-PPA case 15	Certain	59	Unclassifiable	70	Positive		Uncertain	Not sv-PPA	0.53	0.37	0.08
nfv-PPA case 16	Certain	71	nfv-PPA	83	Positive		Uncertain	Not sv-PPA	0.45	0.31	0.06
nfv-PPA case 17	Uncertain	76	nfv-PPA	95	Negative		Uncertain	Not sv-PPA	0.26	0.05	0.07
nfv-PPA case 18	Uncertain	72	Unclassifiable	68	Negative		nfv-PPA	Not sv-PPA	0.17	0.74	0.07
nfv-PPA case 19	Uncertain	69	nfv-PPA	86	Negative		Uncertain	Not sv-PPA	0.44	0.21	0.09
sv-PPA case 1	Certain	68	sv-PPA	52	Negative	FTLD-Tau	Not lv-PPA; Not nfv-PPA	sv-PPA	0.15	0.04	0.80
sv-PPA case 2	Certain	53	sv-PPA	66	Negative		Not lv-PPA; Not nfv-PPA	sv-PPA	0.03	0.02	0.94
sv-PPA case 3	Certain	60	sv-PPA	65	Negative	FTLD-TDP Type C	Not lv-PPA; Not nfv-PPA	sv-PPA	0.08	0.03	0.87
sv-PPA case 4	Certain	53	sv-PPA	61	Negative		Not lv-PPA; Not nfv-PPA	sv-PPA	0.04	0.02	0.94
sv-PPA case 5	Certain	69	sv-PPA	46	Negative	FTLD-TDP Type C	Uncertain	Not sv-PPA	0.25	0.06	0.69
sv-PPA case 6	Certain	63	sv-PPA	64	Negative		Not lv-PPA; Not nfv-PPA	sv-PPA	0.10	0.07	0.83
sv-PPA case 7	Certain	65	Not available	60	Negative		Not lv-PPA; Not nfv-PPA	sv-PPA	0.04	0.03	0.93
sv-PPA case 8	Certain	58	Not available	44	Negative		Not lv-PPA; Not nfv-PPA	sv-PPA	0.19	0.01	0.79
sv-PPA (right) case 9	Certain	73	sv-PPA	74	Negative		Uncertain	Not sv-PPA	0.09	0.12	0.71

sv-PPA case 10	Uncertain	81	Not available	58	N/A	FTLD-TDP Type C	Not lv-PPA; Not nfv-PPA	sv-PPA	0.06	0.04	0.89
sv-PPA case 11	Uncertain	71	sv-PPA	48	Negative		Not lv-PPA; Not nfv-PPA	sv-PPA	0.11	0.06	0.83
sv-PPA case 12	Uncertain	75	lv-PPA	84	Positive		Uncertain	Not sv-PPA	0.10	0.12	0.54
sv-PPA case 13	Certain	57	sv-PPA	62	Positive		Not lv-PPA; Not nfv-PPA	sv-PPA	0.06	0.02	0.91

Note: all PPA patients completed the Addenbrooke’s Cognitive Examination-Revised (ACE-R). Details on how ACE-R performance scores were converted to equivalent ACE-III scores are reported in Supplementary Table 7. Patients below the black line (e.g. lv-PPA cases 14–17) were deemed atypical due to either the neurologist’s clinical opinion or atypical findings on PiB-PET or brain autopsy. ^ Patient 12 was included in the initial ACE-III evaluation and modelled data (i.e. Phase 1) but was also included in Phase 2 based on the recent availability of autopsy findings. A more comprehensive version of this table with colour shadings is presented in Supplementary Table 6. * = co-occurring Lewy body pathology; ACE-III = Addenbrooke’s Cognitive Examination-Third edition; ACE-R = Addenbrooke’s Cognitive Examination-Revised; CBS = corticobasal syndrome; Dx according to the neurologist = the most up-to-date clinical diagnosis according to the neurologist based on the patients’ clinical history, brain imaging and most recent clinical and neuropsychological assessment; FTLD = frontotemporal lobar degeneration; lv-PPA = logopenic variant of primary progressive aphasia; nfv-PPA = non-fluent variant of primary progressive aphasia; PALS = Progressive Aphasia Language Scale; PiB-PET = [11C] Pittsburgh compound B positron emission tomography; PSP = progressive supranuclear palsy; sv-PPA = semantic variant of primary progressive aphasia; tau^1^ = corticobasal degeneration type of tau pathology; tau^2^ = globular glial tauopathy; tau^3^ = progressive supranuclear palsy type of tau pathology; tau^4^ = Pick’s type of tau pathology; TDP-43 = transactive response DNA-binding protein-43.

#### Verification of the Addenbrooke’s Cognitive Examination III primary progressive aphasia diagnostic calculator in Alzheimer’s disease patients (Phase 2 Step 4)

Twenty-two of the 68 Alzheimer’s disease patients (32%) were misclassified with PPA (Supplementary Table 8). Importantly, 86.4% (i.e. 19/22) of these patients were misclassified with lv-PPA, i.e. falling within the same neuropathological entity, with only one misclassified as nfv-PPA and two as sv-PPA. For the remaining Alzheimer’s disease patients (*n* = 46), the calculator rejected a diagnostic classification of PPA; incidentally, diagnosis probability values for these patients mostly pointed toward lv-PPA rather than toward nfv-PPA or sv-PPA.

## Discussion

The ACE-III PPA diagnostic calculator demonstrates promising accuracy as a tool to differentiate the PPA variants based on a patient’s item-by-item ACE-III profile. By evaluating the inter-relationships across the ACE-III test items, this clinical tool provides a diagnostic probability value and categorical classification for the individual patient. Overall, the calculator demonstrates encouraging precision, with classification sensitivity for the clinical diagnosis of PPA variants ranging between 100% (i.e. sv-PPA) and 79.9% (i.e. lv-PPA). Verification of the calculator in a separate, neuropathologically confirmed PPA sample further revealed high classification accuracy in sv-PPA and promising trends in lv-PPA and nfv-PPA. The variable rates of accuracy among PPA variants likely reflect the typical diagnostic challenges in brief clinical assessment and the nosological continuity between lv-PPA and nfv-PPA. Taken together, the findings from this study provide new insights into the distinct cognitive profiles of the PPA variants.

### Integrity of the Addenbrooke’s Cognitive Examination III primary progressive aphasia diagnostic calculator

Using multinomial logistic regression techniques, we created an interactive ACE-III PPA diagnostic calculator that evaluates the inter-relationships among ACE-III test items at the individual patient level. The accuracy of the calculator was determined based on two techniques: (i) cross-validation of cases in the model (Phase 1) and (ii) verification in a separate group of *in vivo* amyloid and/or autopsy-confirmed PPA patients (Phase 2). For reasons outlined below, we believe the cross-validation findings provide the best estimates of the calculator’s accuracy. Consistent with previous automated PPA classification tools,^42,43^ the calculator was more sensitive at classifying sv-PPA (100%) than nfv-PPA (80.6%) and lv-PPA (79.9%). While not systematically verified in the present study, these sensitivity rates likely reflect the level of diagnostic certainty experienced by clinicians after a brief consultation and completion of the ACE-III. In this regard, we recommend that the ACE-III PPA diagnostic calculator be used in a similar manner that is as a ‘first-step’ probability screening tool to guide clinicians in their hypothesis-driven testing. In keeping with this premise, we intentionally set the calculator’s classification thresholds (*α*) at conservative (i.e. higher) levels for each PPA variant to encourage the interpretation of the probability values over the calculator’s categorical classification function and to minimise misclassification errors (i.e. false positives). Accordingly, correct categorical classifications in the present study may appear low.

Verification of the calculator in *in vivo* amyloid and/or autopsy-confirmed PPA patients indicated the calculator correctly classified 77% sv-PPA, 16% nfv-PPA, and 24% of lv-PPA patients. Notably, for patients who did not reach a statistical threshold for diagnostic classification, probability values mostly pointed toward the correct diagnosis (Table 2). While the classification rates of the sv-PPA patients were encouraging, accurate classification of lv-PPA and nfv-PPA patients were lower than expected. It is possible that the relatively small sample sizes, inclusion of atypical patients (i.e. patients with inconsistent clinical and/or pathological findings), and/or the conversion of ACE-R to ACE-III scores in this cohort contributed to the lower accuracy rates. These observations notwithstanding, the accuracy rates across variants somewhat reflected that observed in the cross-validation modelling. That is, the cognitive and language features of lv-PPA and nfv-PPA are more heterogenous than sv-PPA and that the calculator is less effective at capturing these variances.^5,16,19,42^ Another, and not mutually exclusive, possibility is that the language features of lv-PPA and nfv-PPA overlap considerably^2,4,13,42^ and that the calculator is less able to differentiate these syndromes.

### Language and cognitive profiles on the Addenbrooke’s Cognitive Examination III

A secondary aim of this study was to evaluate the language and cognitive profiles of the PPA variants based on their ACE-III item-by-item scores (i.e. Phase 1 Step 1). We demonstrate that ACE-III profiles among the PPA variants are more salient when evaluating item-by-item scores than aggregate scores at the subdomain or overall (i.e. Total) ACE-III level. This is not surprising given each item within the ACE-III is purported to measure discrete abilities (which pertain to an overarching construct, such as Attention or Memory for example).^26^ Understanding how these item-specific scores vary across the PPA variants is necessary to interpret the output from the ACE-III PPA diagnostic calculator. Accordingly, we discuss below the critical findings regarding the language and cognitive profiles of the PPA variants based on the ACE-III test items.

#### Language profiles

This study found that performance on the language items on the ACE-III reflected the characteristic language profiles of each PPA variant. Specifically, the sv-PPA group (from Phase 1) displayed prominent anomia (i.e. confrontation naming) and word comprehension (i.e. object comprehension, reading) deficits in the context of preserved speech fluency (i.e. sentence repetition), syntax (i.e. three commands) and word repetition (i.e. single-word repetition). In contrast, nfv-PPA patients presented with effortful speech (i.e. single word and sentence repetition) in the context of relatively preserved word comprehension (i.e. object comprehension, reading) and object knowledge (i.e. mild confrontation naming problems). Finally, the lv-PPA group displayed deficits in word retrieval (i.e. confrontation naming) and sentence repetition (i.e. relative to single-word repetition), with word comprehension relatively preserved (i.e. object comprehension). Put together, we demonstrate that the ACE-III, at an item-by-item level, effectively elicits the distinct language deficits among PPA variants.

#### Cognitive profiles beyond language

Consistent with previous research, we found that the cognitive profiles of the PPA variants are distinct and extend beyond the primary domain of language.^14,15,20^ Our findings confirm that lv-PPA display widespread, diffuse deficits across all domains on the ACE-III. By contrast, the cognitive profiles of nfv-PPA and sv-PPA are more circumscribed, with difficulties largely centred on language and verbal fluency, and with aspects of attention (in sv-PPA), memory (in nfv-PPA), and visuospatial ability (in both nfv-PPA and sv-PPA) remaining less affected.

Visuospatial abilities in PPA have received increased attention in the literature, with recent studies suggesting visuoconstructional skills are particularly compromised in lv-PPA.^14,20,21^ While limited in scope (i.e. narrow range of scores, small selection of items), our study suggests that the clock drawing item was more effective at differentiating lv-PPA from the other variants than the copy and/or visuoperceptual items. Notably, while not as prominent as lv-PPA, visuospatial proficiency in nfv-PPA was also diminished (i.e. Visuospatial subdomain: nfv-PPA < controls). Taken together, our findings contribute to a growing body of research demonstrating a gradient of visuospatial impairment across the PPA variants, ranging from relatively spared in sv-PPA to grossly compromised in lv-PPA, and with nfv-PPA lying in between these two extremes.

Turning our attention briefly to the other subdomains, all PPA variants displayed impairments on aspects of attention, verbal fluency, and verbal memory. Contamination of language ability, however, likely contributed to performance scores on many of these items. These observations notwithstanding, the lv-PPA group demonstrated particular difficulty with verbal learning and the lv-PPA and sv-PPA groups demonstrated particular difficulty with long-term retention of verbal information.^14,15,44–46^ Evidence from other studies suggests that the learning and memory difficulties in lv-PPA extends to the non-verbal domain (suggesting an inherent problem with the memory system), whereas non-verbal memory in sv-PPA remains relatively spared (suggesting their difficulties are intertwined with language ability).^21,24,44–46^ Finally, while our previous study demonstrates that the overall ACE-III Attention subdomain remains relatively spared in nfv-PPA over the disease course,^20^ this study suggests that some aspects of attentional control may be compromised. Specifically, the nfv-PPA cohort demonstrated impaired performance on the serial 7 s task at baseline assessment. Further, and interestingly, the nfv-PPA cohort demonstrated greater difficulty with counting dots than the other PPA variants. While this latter finding may appear to relate to visuospatial disturbance, we question if an attentional component may be at play. Previous studies suggest that aspects of attentional control are compromised in nfv-PPA, especially on measures that require updating and/or manipulating information in mind (i.e. working memory).^15,24,47^ Most items on the ACE-III Attention subdomain do not index executive control processes in depth (i.e. orientation, three-word registration) and thus, we propose that higher-level attentional skills in nfv-PPA may be more problematic than their overall attentional profiles on the ACE-III may suggest.

From a clinical perspective, our findings demonstrate that the ACE-III should only serve as a brief screening tool in the diagnosis of PPA and that clinicians need to supplement the assessment with additional non-verbal memory and attentional measures. Further, the interpretation of the ACE-III is undoubtedly improved when clinicians consider other qualitative aspects (such as error types, language and/or behavioural features) that may (or may not) interact with the patient’s performance profile.

### Considerations and further applications of the Addenbrooke’s Cognitive Examination III primary progressive aphasia diagnostic calculator

A few caveats are warranted when using the ACE-III PPA diagnostic calculator. The calculator was built based on data from an Australian population with relatively high levels of education and English proficiency. We therefore caution the use of this tool in non-English speaking countries, in culturally and linguistically diverse populations, and/or in individuals with limited formal education. The PPA cohort used to build the calculator largely scored between the mid-60 s to low-70 s on the ACE-III Total score and had, on average, 3 to 5 years of reported symptoms. While the calculator was verified across a range of performance scores (PPA ACE-III scores between 40 and 95/100), we caution its use in patients who score exceedingly well (i.e. above 88/100) or poorly (i.e. below 45/100). Finally, as demonstrated by our verification in typical Alzheimer’s disease (Phase 2 Step 4), we only recommend that the calculator be used in patients with a working diagnosis of PPA. Misuse of the calculator in populations other than PPA may result in misclassification errors.

While strict diagnostic criteria were used for the PPA patients included in the building of the calculator (i.e. clinical, cognitive, and imaging information sufficient to establish a clinical diagnosis of a PPA syndrome), pathological confirmation of these patients were unknown. Accordingly, the calculator makes no claim in predicting underlying pathology. In this regard, we note that the neuropathologically substantiated PPA sample population (Phase 2 Step 4; Table 2) largely reflected the pathological proportions typically reported in the literature: ∼75% of lv-PPA patients have underlying Alzheimer pathology, ∼65% of nfv-PPA have TDP-43 or tauopathy, and ∼80% of sv-PPA have TDP−43.^6–9,48^ The presence of other pathological findings in our cohort (i.e. lv-PPA case 13 with FTLD-Tau, sv-PPA case 1 with FTLD-Tau) underscores the fact that the clinico-pathological correspondence in PPA remains far from absolute.^49^ More research is warranted to determine if specific cognitive profiles within PPA syndromes can predict underlying pathology.

Lastly, a proportion of nfv-PPA patients will develop parkinsonian features (e.g. limb apraxia, akinesia/bradykinesia, motor rigidity) as the disease progresses (typically in the moderate to severe disease stages).^50,51^ In the present study, we excluded patients presenting with a clear or suspected parkinsonian syndrome for two reasons. First, we wanted our calculator to align with the current PPA consensus criteria (i.e. ‘a clear parkinsonian syndrome should not be present at the time of diagnosis’).^3(p.1008)^ Second, we assumed that nfv-PPA patients with suspected or clear parkinsonism would be more easily distinguishable from the other PPA variants based on their clinical profiles alone (as parkinsonian features are not common in lv-PPA or sv-PPA).^50^ Accordingly, we anticipate that our calculator could be particularly useful at distinguishing PPA patients with no or only mild motor features. We acknowledge, however, that this may not reflect the more nuanced motor presentations in the clinical setting. Given nfv-PPA patients with parkinsonian features likely perform poorly on visuospatial related tasks due to their inherent motor dysfunction^52–54^ (a feature not evaluated in the present study), we caution the calculator’s use in nfv-PPA patients with marked motor features. Future research is needed to delineate the respective cognitive profiles of nfv-PPA patients with and without concomitant parkinsonian features.

## Conclusions

The ACE-III PPA diagnostic calculator provides a promising adjunct to the ACE-III. It is the first interactive clinical tool to evaluate the inter-relationships across ACE-III test items to predict a PPA variant. The ACE-III PPA diagnostic calculator represents a new frontier in the automatic subtyping of PPA variants and the evaluation of cognitive domains beyond language to improve diagnosis.

## Supplementary Material

fcac161_Supplementary_Data

## Abbreviations

ACE-III =: Addenbrooke’s Cognitive Examination-Third edition
ACE-R =: Addenbrooke’s Cognitive Examination-Revised
ANOVA =: analysis of variance
CBS =: corticobasal syndrome
DAD =: Disability Assessment for Dementia
FTLD =: frontotemporal lobar degeneration
lv-PPA =: logopenic variant of primary progressive aphasia
nfv-PPA =: non-fluent variant of primary progressive aphasia
PALS =: Progressive Aphasia Language Scale
PiB-PET =: [11C] Pittsburgh compound B PET
PSP =: progressive supranuclear palsy
SPSS =: IBM Statistical Package for the Social Sciences
sv-PPA =: semantic variant of primary progressive aphasia
TDP-43 =: transactive response DNA-binding protein-43.
