# Supplementary material for: Utility of the Addenbrooke’s Cognitive Examination III online calculator to differentiate the primary progressive aphasia variants
Source: Brain Commun. 2022 Jul 7;4(4):fcac161. doi: 10.1093/braincomms/fcac161 (PMC9336588; doi:10.1093/braincomms/fcac161)
Supplement: fcac161_Supplementary_Data [file fcac161_Supplementary_Data.pdf]

Supplementary Material

Utility of the Addenbrooke's Cognitive Examination-III online calculator to differentiate the primary progressive aphasia variants

David Foxe*, Anne Hu*, Sau Chi Cheung, Rebekah M. Ahmed, Nicholas J. Cordato, Emma Devenney, Yun Tae Hwang, Glenda M. Halliday, Nicole Mueller, Cristian E. Leyton, John R. Hodges, James R. Burrell, Muireann Irish, Olivier Piguet

*These authors contributed equally to the work

METHODS

Participants

The current consensus criteria states that 'a clear parkinsonian syndrome (rigidity, tremor) 'should not be present at the time of diagnosis, although mild limb apraxia and difficulty with fine finger movements can be noted.'[1(p.1008)] The consensus criteria, however, do not elaborate on the definition of 'clear'. Accordingly, in the current study, we took a precautionary approach and excluded patients with frank parkinsonism and/or patients with suspected parkinsonian or motor neuron features. Supplementary Figure 1 provides a comprehensive illustration of the parkinsonian and motor neuron profiles of nfv-PPA patients i) included and ii) excluded in Phase 1 of this study.

Neuropsychological tests routinely administered to PPA patients and controls at the FRONTIER Research Group clinic

i) Digit Span Forward and Backward subtests of the Wechsler Memory Scale–Third edition WMS-III),[2] ii) Rey Complex Figure Test Copy and Recall (RCFT),[3] iii) Sydney Language Battery (SYDBAT), which includes measures of confrontation naming, single-word repetition, word comprehension and semantic association,[4] iv) verbal letter fluency (with letters F, A and S),[5] v) the Sentence Repetition

task from the Multilingual Aphasia Examination,[6] vi) Trail Making Test, [7] and, vii) the Face and Emotion Processing Battery.[8,9]

Results

Demographics

Overall group differences were present for all baseline ACE-III subdomain raw scores (all p values < .001): Attention $F(3, 190) = 45.049$; Memory $F(3, 190) = 88.127$; Verbal Fluency $F(3, 190) = 169.899$; Language $F(3, 190) = 136.932$; Visuospatial $F(3, 190) = 31.452$ (Table 1). Compared to Controls, patient groups were impaired on all ACE-III subdomains (all p values $\leq$ .001), with the exception of the sv-PPA group which showed relatively intact performance on the Visuospatial subdomain (p = .162).

Direct comparisons of the patient groups on the baseline ACE-III subdomain raw scores are presented in Table 1. On the Attention subdomain, the lv-PPA group were more impaired than the nfv-PPA groups (p values $\leq$ .003), whereas no differences were observed between the nfv-PPA and sv-PPA groups on this measure (p = .418). On the Memory subdomain, the lv-PPA and sv-PPA groups performed worse than the nfv-PPA group (p values < .001). Performances among the lv-PPA and sv-PPA on this measure did not differ significantly (p = .212). On the Fluency subdomain, the nfv-PPA were more impaired than the sv-PPA group (p < .001). Performances did not differ on this measure among the lv-PPA and nfv-PPA group (p = .593) or among the lv-PPA and sv-PPA groups (p = .065). The lv-PPA and nfv-PPA groups were similarly impaired on the Language (p = 1.000) but less impaired than the sv-PPA group (p values < .001). Lastly, on the Visuospatial subdomain, the lv-PPA and nfv-PPA groups were more impaired than the sv-PPA group (p values $\leq$ .002). Performances on this measure did not differ significantly between the lv-PPA and nfv-PPA groups (p = .461).

ACE-III items

The PPA groups showed distinct ACE-III profiles at the item-by-item level. Relative to controls, the PPA groups were impaired on most ACE-III items. Notable exceptions across PPA groups were, however, present. The sv-PPA group did not differ significantly from controls on all visuospatial items (i.e., loops, cube, clock, dot counting, fragmented letters), most repetition items (i.e., single words, 'glitters' sentence), three step commands, and on several attention items (orientation to time, three-word registration/learning, serial 7s). Relative to controls, the nfv-PPA group's performance was spared on aspects of visuospatial ability (i.e., clock drawing, fragmented letters), semantic association, and on free recall of a name and address. The lv-PPA group did not differ significantly from controls on basic visuoperceptual skills (i.e., dot counting, fragmented letters) and the semantic association item.

Direct comparisons of the PPA groups revealed cognitive profiles characteristic of each variant. Comparison of the two non-fluent variants (i.e., lv-PPA, nfv-PPA) revealed the lv-PPA group were disproportionately impaired on the name and address learning item and clock drawing, whereas the nfv-PPA group were disproportionately impaired on single word repetition and dot counting. Relative to the non-fluent groups, the sv-PPA were more impaired on retrograde memory, confrontation naming, semantic knowledge (i.e., word-picture matching) and reading, and were additionally more impaired than the nfv-PPA group on orientation to location (i.e., geography), and aspects of memory (i.e., three-word recall, free memory recall of name and address). By contrast, the sv-PPA group performed better than the non-fluent groups on aspects of attention (i.e., orientation to time, serial 7s), repetition (i.e., single word, sentences) and visuospatial abilities (i.e., loops, cube); the sv-PPA group were also superior to the nfv-PPA group on letter fluency, writing and dot counting, and superior to lv-PPA on clock drawing.

Supplementary Table 1. Progressive aphasia language scale (PALS) scoring criteria

Item	Score			
	0 (None)	1 (Questionable)	2 (Definite)	3 (Severe)
**Spontaneous Speech**				
Motor speech disorders	No evidence	Isolated and inconsistent articulatory errors. Groping speech is occasionally present.	Consistent or evident distortions, especially for less common polysyllabic words, leading to evident slurred speech. Loss of prosody is often present.	Speech is continuously distorted or slurred, often causing unintelligible pronunciation or slow output. Consistently abnormal prosody, rate of speech, and resolution of utterances
Agrammatism	No evidence	Avoidance of complex grammatical structures. Syntax and word inflection are largely preserved. There are only isolated and questionable errors.	Consistent oversimplification of sentence structures, with evidence of grammatical errors, especially for syntax and verb conjugation (i.e., inflection). Closed-type words (e.g., prepositions, auxiliary verbs, or articles) are omitted or incorrectly used.	Frequent omission of closed-type words while open-type words are often incorrectly inflected. Language production is telegraphic, with evident oversimplification of sentence structure.
**Tasks**				
Naming	No anomia	Nearly normal with just one or two naming errors or omissions, usually for uncommon items (e.g., *tongs, rhinoceros*, etc.). Frequent and marked self-corrections, subtle delays in retrieving names, or naming doubtfully should be included in this level.	Although may correctly name some well-known items, there is consistent and unmistakable naming errors.	Just able to correctly name a few well-known items (less than a third of items), with marked delays in retrieving names.
Single word repetition	Flawless repetition	Occasional (1-2) and subtle errors mainly due to mispronunciation or word fragmentation, involving only longer, or more complex words.	Evident and consistent mistakes due to mispronunciation, omissions, substitutions, phoneme reversal or word fragmentation.	Frequent and evident errors. Only able to repeat the simpler-more-common words (e.g., *banana, potato*) or one to two syllables of the longer-more-complex words.
Single word comprehension	Normal comprehension	Low frequency words (e.g., *ferocious, indigenous*) are occasionally unknown. In general, most words are somehow correctly defined, pointed to, or related to their concept.	Marked impairment in defining or object-word matching, able to provide broad superordinate category (e.g., *an animal*), but not able to elaborate on details. Preservation of more familiar items.	Only a few highly familiar words might be correctly recognised, with consistent failures in pointing to, or matching well-known items, such as *tiger* or *crocodile*.
Sentence repetition	Flawless repetition	Subtle mistakes due to word errors, self-corrections, or long latency. Although minimal omissions may be present, there is frequent repetition of t the main sentence's components.	Consistent errors (namely disorganisation, omissions, or mispronunciation) when the patient tries to repeat longer sequences (e.g., *The Chinese fan*…).	Unable to correctly repeat phrases with more than two short words.
Sentence comprehension	Normal comprehension	Occasional or minimal mistakes. Longer latency in replying, asking for instructions to be repeated, and self-corrections should be considered.	Consistent mistakes involving complex instructions.	Marked and consistent mistakes in direct instructions (*e.g., touch the pen then the toothbrush*).

Supplementary Table 2. Terms used in this study and in the online PPA ACE-III diagnostic calculator

Terms used in Figures and Tables	Terms used in the PPA ACE-III calculator
Attention_Time	Attention: Orientation to Time (0-5)
Attention_Geography	Attention: Orientation to Location (0-5)
Attention_3_Word_Registration	Attention: Registration of Three Words (0-3)
Attention_Serial_7s	Attention: Serial 7 Subtraction (0-5)
Memory_3_Word_Recall	Memory: Recall of Three Words (0-3)
Fluency_Letter_Score	Verbal Fluency: Letter Score (0-7)
Fluency_Animal_Score	Verbal Fluency: Animal Score (0-7)
Memory_Learning_Address	Memory: Learning of Name and Address (0-7)
Memory_Retrograde	Memory: Retrograde (i.e., Prime Minister etc.) (0-4)
Language_3_Commands	Language: Comprehension (pencil and paper) (0-3)
Language_Writing	Language: Sentence Writing (0-2)
Language_Repetition_Single_Words	Language: Word Repetition ('caterpillar' etc.) (0-2)
Language_Repetition_Glitters	Language: Sentence Repetition 1 ('All that glitters…') (0-1)
Language_Repetition_A_Stitch	Language: Sentence Repetition 2 ('A stitch in time…') (0-1)
Language_Confrontation_Naming	Language: Object Naming (0-12)
Language_Semantics	Language: Object Comprehension (0-4)
Language_Reading	Language: Reading (0-1)
Visuospatial_Loops	Visuospatial: Intersecting Infinity Loops (0-1)
Visuospatial_Cube	Visuospatial: 3D Wire Cube
Visuospatial_Clock	Visuospatial: Clock Drawing (0-5)
Visuospatial_Dots	Visuospatial: Dot Counting (0-4)
Visuospatial_Letters	Visuospatial: Fragmented Letters (0-4)
Memory_Recall_Address	Memory: Recall of Name and Address (0-7)
Memory_Recognition_Address	Memory: Recognition of Name and Address (0-5)

Supplementary Table 3. Performances on the ACE-III items in a sample of 30 healthy controls and 104 controls

	30 Controls	104 Controls
Sex (m : f)	12:18	49:55
Age (y)	66.8 (8.3)	67 (7.8)
Education (y)	13.7 (2.5)	14.1 (2.9)
ACE-III Total (100)	96.3 (2.6)	95.2 (2.9)
ACE-III Attention (18)	17.5 (0.8)	17.3 (1)
ACE-III Memory (26)	24.7 (1.5)	24.7 (1.4)
ACE-III Fluency (14)	12.8 (1)	12.2 (1.5)
ACE-III Language (26)	25.7 (0.6)	25.5 (0.8)
ACE-III Visuospatial (16)	15.6 (0.6)	15.6 (0.7)
Attention_Time	4.9 (0.3)	4.9 (0.3)
Attention_Geography	4.9 (0.4)	4.9 (0.4)
Attention_3_Word_Registration	2.9 (0.4)	3 (0.2)
Attention_Serial_7s	4.8 (0.4)	4.7 (0.8)
Memory_3_Word_Recall	2.8 (0.4)	2.9 (0.4)
Fluency_Letter_Score	6.5 (0.6)	6.1 (1)
Fluency_Animal_Score	6.3 (0.6)	6.1 (0.9)
Memory_Learning_Address	7 (0)	7 (0.1)
Memory_Retrograde	3.8 (0.4)	3.8 (0.4)
Language_3_Commands	3 (0)	3 (0.3)
Language_Writing	2 (0.2)	1.9 (0.3)
Language_Repetition_Single_Words	2 (0)	1.9 (0.3)
Language_Repetition_Glitters	1 (0)	1 (0)
Language_Repetition_A_Stitch	1 (0)	1 (0.1)
Language_Confrontation_Naming	11.9 (0.3)	11.9 (0.4)
Language_Semantics	3.9 (0.3)	3.9 (0.3)
Language_Reading	0.9 (0.3)	0.9 (0.3)
Visuospatial_Loops	0.9 (0.3)	1 (0.2)
Visuospatial_Cube	1.9 (0.3)	1.9 (0.3)
Visuospatial_Clock	4.8 (0.4)	4.8 (0.5)
Visuospatial_Dots	3.9 (0.3)	3.9 (0.3)
Visuospatial_Letters	4 (0)	4 (0)
Memory_Recall_Address	6.2 (1.1)	6.1 (1)
Memory_Recognition_Address	4.9 (0.3)	4.9 (0.3)

Notes: Values are mean ± standard deviation. The 104 controls were included in the characterisation of PPA performance profiles on the ACE-III (Phase 1 Step 1). The sample of 30 controls were included in the PPA ACE-III diagnostic calculator (Phase 1 Step 2 and 3).

Supplementary Table 4. Defining terms visuospatial, visuoconstructional and visuoperceptual

Visuospatial	Broad term relating to visual and spatial abilities. The visuospatial ACE-III subdomain incorporates measures of visuoconstruction and visuoperception
Visuoconstruction	Involves the ability to organise and manually manipulate visual and/or spatial information to make a design (i.e., drawing). Visuoconstruction items on the ACE-III includes drawing the infinity loops, 3-dimensional cube, and clock
Visuoperceptual	Involves recognising objects based on their form, pattern, and colour. Visuoperceptual items on the ACE-III includes counting dots and identifying fragmented letters

Supplementary Table 5. Performances on the ACE-III items in primary progressive aphasia patients (n = 90) and healthy controls (n = 104)

	lv-PPA	nfv-PPA	sv-PPA	Controls	H or χ^2 value	*p*	Post hoc test
Attention_Time	4.1 (1.3)	4.4 (0.8)	4.9 (0.3)	4.9 (0.3)	34.988	< .001	lv-PPA, nfv-PPA < sv-PPA, Controls
Attention_Geography	3.8 (1)	4.4 (0.7)	3.7 (0.9)	4.9 (0.4)	73.169	< .001	sv-PPA < nfv-PPA; PPA < Controls
Attention_3_Word_Registration	2.7 (0.7)	2.8 (0.4)	3 (0.2)	3 (0.2)	12.884	.005	lv-PPA, nfv-PPA < Controls
Attention_Serial_7s	2.5 (2)	3.4 (1.8)	4.4 (1.2)	4.7 (0.8)	46.296	< .001	lv-PPA, nfv-PPA < sv-PPA, Controls
Memory_3_Word_Recall	1.8 (1)	2.4 (0.7)	1.3 (1.2)	2.9 (0.4)	75.458	< .001	sv-PPA < nfv-PPA; PPA < Controls
Fluency_Letter_Score	2.8 (1.9)	1.7 (1.3)	3.9 (1.5)	6.1 (1)	116.636	< .001	nfv-PPA < sv-PPA; PPA < Controls
Fluency_Animal_Score	2.1 (1.6)	2.3 (1.4)	2.4 (2)	6.1 (0.9)	131.046	< .001	PPA < Controls
Memory_Learning_Address	4.8 (2.1)	6.1 (1.7)	6.2 (1.3)	7 (0.1)	74.627	< .001	lv-PPA < nfv-PPA, sv-PPA < Controls
Memory_Retrograde	2.3 (1.4)	2.5 (1.3)	1.2 (1.2)	3.8 (0.4)	104.629	< .001	sv-PPA < lv-PPA, nfv-PPA < Controls
Language_3_Commands	2.3 (1.1)	2.3 (0.9)	2.9 (0.3)	3 (0.3)	49.562	< .001	lv-PPA, nfv-PPA < sv-PPA, Controls
Language_Writing	1.3 (0.8)	0.8 (0.7)	1.5 (0.7)	1.9 (0.3)	66.989	< .001	nfv-PPA < sv-PPA; PPA < Controls
Language_Repetition_Single_Words	1.1 (1)	0.4 (0.8)	1.8 (0.4)	1.9 (0.3)	85.147	< .001	nfv-PPA < lv-PPA < sv-PPA, Controls
Language_Repetition_Glitters	0.8 (0.4)	0.5 (0.5)	1 (0.2)	1 (0)	65.007^	< .001	nfv-PPA < sv-PPA; lv-PPA, nfv-PPA < Controls
Language_Repetition_A_Stitch	0.5 (0.5)	0.5 (0.5)	0.9 (0.3)	1 (0.1)	61.702^	< .001	lv-PPA, nfv-PPA < sv-PPA < Controls
Language_Confrontation_Naming	9.6 (3.1)	11 (1.3)	4.4 (2.9)	11.9 (0.4)	121.413	< .001	sv-PPA < lv-PPA, nfv-PPA < Controls
Language_Semantics	3.5 (0.9)	3.6 (0.7)	1.6 (1.4)	3.9 (0.3)	93.11	< .001	sv-PPA < lv-PPA, nfv-PPA, Controls
Language_Reading	0.6 (0.5)	0.4 (0.5)	0.1 (0.3)	0.9 (0.3)	84.599^	< .001	sv-PPA < lv-PPA; PPA < Controls
Visuospatial_Loops	0.6 (0.5)	0.6 (0.5)	0.9 (0.3)	1 (0.2)	38.151^	< .001	lv-PPA, nfv-PPA < sv-PPA, Controls
Visuospatial_Cube	1.4 (0.8)	1.4 (0.7)	1.9 (0.4)	1.9 (0.3)	35.615	< .001	lv-PPA, nfv-PPA < sv-PPA, Controls
Visuospatial_Clock	3.2 (1.3)	4.2 (1.1)	4.2 (1.2)	4.8 (0.5)	54.085	< .001	lv-PPA < nfv-PPA, sv-PPA, Controls
Visuospatial_Dots	3.7 (0.7)	3.3 (1)	3.9 (0.3)	3.9 (0.3)	22.958	< .001	nfv-PPA < lv-PPA, sv-PPA, Controls
Visuospatial_Letters	4 (0.2)	4 (0.2)	4 (0)	4 (0)	5.372	.146	Nil
Memory_Recall_Address	3.1 (2.4)	4.8 (2.4)	1.7 (1.9)	6.1 (1)	91.474	< .001	sv-PPA < nfv-PPA; sv-PPA, lv-PPA < Controls
Memory_Recognition_Address	4.2 (1)	4.3 (1.1)	3.8 (1.2)	4.9 (0.3)	47.646	< .001	PPA < Controls

Notes: Values are mean ± standard deviation. ^ + χ^2 test.

Supplementary Table 6. Application of the Addenbrooke's Cognitive Examination-III (ACE-III) primary progressive aphasia diagnostic calculator in PiB-PET or autopsy-confirmed patients

Patient (Dx according to neurologist)	Dx certainty	Differential Dx	Sex	Education (y)	Age at PALS and/or ACE-R	PALS diagnosis	ACE-R Total score	PiB-PET result	Brain pathology	ACE-III PPA diagnostic calculator				
										lv-PPA vs. nfv-PPA calculator prediction	sv-PPA vs. non sv-PPA calculator prediction	lv-PPA probability	nfv-PPA probability	sv-PPA probability
lv-PPA case 1	Certain		Male	19	68	lv-PPA	72	Positive	AD*	Uncertain	Not sv-PPA	0.46	0.22	0.24
lv-PPA case 2	Certain		Female	16	73	Unclassifiable	69	Positive		**lv-PPA**	Not sv-PPA	0.73	0.12	0.10
lv-PPA case 3	Certain		Female	18	56	lv-PPA	58	Positive		Uncertain	Not sv-PPA	0.70	0.08	0.21
lv-PPA case 4	Certain		Male	11.5	72	lv-PPA	74	Positive		Uncertain	Not sv-PPA	0.36	0.13	0.45
lv-PPA case 5	Certain		Male	13.5	59	lv-PPA	63	Positive		Uncertain	Not sv-PPA	0.39	0.11	0.48
lv-PPA case 6	Certain		Male	12	61	nfv-PPA	58	Positive	AD	Uncertain	Not sv-PPA	0.52	0.41	0.05
lv-PPA case 7	Certain		Female	15	69	lv-PPA	55	Positive		**lv-PPA**	Not sv-PPA	0.87	0.07	0.05
lv-PPA case 8	Certain		Female	9	77	Unclassifiable	90	Positive		Uncertain	Not sv-PPA	0.23	0.40	0.04
lv-PPA case 9	Certain		Female	11	63	Unclassifiable	56	Positive		Uncertain	Not sv-PPA	0.32	0.30	0.36
lv-PPA case 10	Certain		Female	12	75	Not available	44	N/A	AD*	Uncertain	Not sv-PPA	0.63	0.23	0.15
lv-PPA case 11	Certain		Female	18	53	lv-PPA	68	Positive		Uncertain	Not sv-PPA	0.36	0.25	0.38
lv-PPA case 12	Certain		Male	12	59	lv-PPA	63	Positive		Uncertain	Not sv-PPA	0.36	0.22	0.39
lv-PPA case 13	Certain		Male	11	70	nfv-PPA	74	Positive	FTLD-Tau[1]	**lv-PPA**	Not sv-PPA	0.73	0.04	0.13
lv-PPA case 14	Uncertain	Atypical AD - Aphasic	Male	12	65	lv-PPA	44	Positive		**lv-PPA**	Not sv-PPA	0.94	0.04	0.03
lv-PPA case 15	Uncertain	Atypical AD - Aphasic	Female	11	71	Unclassifiable	64	Positive		Uncertain	Not sv-PPA	0.40	0.19	0.39
lv-PPA case 16	Uncertain	sv-PPA	Female	16	65	lv-PPA	60	Equivocal	AD	Not lv-PPA; Not nfv-PPA	**sv-PPA**	0.11	0.02	0.86
lv-PPA case 17	Uncertain	nfv-PPA	Female	14	65	Unclassifiable	75	Negative		Uncertain	Not sv-PPA	0.60	0.14	0.17
nfv-PPA case 1	Certain		Male	17.5	55	nfv-PPA	62	Negative		Uncertain	Not sv-PPA	0.31	0.48	0.19
nfv-PPA case 2	Certain		Female	8	80	Not available	41	N/A	FTLD-Tau[2]	Uncertain	Not sv-PPA	0.54	0.43	0.00
nfv-PPA case 3	Certain		Male	9	78	nfv-PPA	56	Equivocal	FTLD-Tau[3]*	Uncertain	Not sv-PPA	0.35	0.63	0.01
nfv-PPA case 4	Certain		Female	9	71	Unclassifiable	83	Negative	FTLD-Tau[3]	Uncertain	Not sv-PPA	0.20	0.39	0.20
nfv-PPA case 5	Certain		Male	15	47	nfv-PPA	87	Negative	FTLD-Tau[1]*	Uncertain	Not sv-PPA	0.24	0.53	0.05

nfv-PPA case 6	Certain	Pseudobulbar palsy	Female	11	70	nfv-PPA	89	Negative		Uncertain	Not sv-PPA	0.21	0.52	0.03
nfv-PPA case 7	Certain		Female	12.5	72	Not available	75	Negative	FTLD-Tau[1]*	**nfv-PPA**	Not sv-PPA	0.08	0.86	0.03
nfv-PPA case 8	Certain		Male	14	77	Not available	74	Negative		**nfv-PPA**	Not sv-PPA	0.13	0.74	0.11
nfv-PPA case 9	Certain		Male	15	73	Not available	87	Negative		Uncertain	Not sv-PPA	0.36	0.26	0.19
nfv-PPA case 10	Certain		Female	14	60	Not available	85	Negative	FTLD-Tau	Uncertain	Not sv-PPA	0.14	0.63	0.11
nfv-PPA case 11	Certain	Pure apraxia of speech	Female	15	70	nfv-PPA	85	Negative	FTLD-Tau[4]	Uncertain	Not sv-PPA	0.34	0.40	0.04
nfv-PPA case 12^	Certain		Female	15	63	Not available	74	Negative	FTLD-Tau	Uncertain	Not sv-PPA	0.39	0.52	0.05
nfv-PPA case 13	Uncertain	CBS	Male	8	71	Not available	45	Equivocal		Uncertain	Not sv-PPA	0.68	0.30	0.02
nfv-PPA case 14	Uncertain	lv-PPA	Male	12	70	Not available	71	Negative		Uncertain	Not sv-PPA	0.30	0.57	0.11
nfv-PPA case 15	Certain		Male	15.5	59	Unclassifiable	70	Positive		Uncertain	Not sv-PPA	0.53	0.37	0.08
nfv-PPA case 16	Certain		Male	9	71	nfv-PPA	83	Positive		Uncertain	Not sv-PPA	0.45	0.31	0.06
nfv-PPA case 17	Uncertain	PSP	Male	18	76	nfv-PPA	95	Negative		Uncertain	Not sv-PPA	0.26	0.05	0.07
nfv-PPA case 18	Uncertain	lv-PPA	Female	9	72	Unclassifiable	68	Negative		**nfv-PPA**	Not sv-PPA	0.17	0.74	0.07
nfv-PPA case 19	Uncertain	PSP	Female	11	69	nfv-PPA	86	Negative		Uncertain	Not sv-PPA	0.44	0.21	0.09
sv-PPA case 1	Certain		Female	9	68	sv-PPA	52	Negative	FTLD-Tau	Not lv-PPA; Not nfv-PPA	**sv-PPA**	0.15	0.04	0.80
sv-PPA case 2	Certain		Male	19	53	sv-PPA	66	Negative		Not lv-PPA; Not nfv-PPA	**sv-PPA**	0.03	0.02	0.94
sv-PPA case 3	Certain		Male	11.5	60	sv-PPA	65	Negative	FTLD-TDP Type C	Not lv-PPA; Not nfv-PPA	**sv-PPA**	0.08	0.03	0.87
sv-PPA case 4	Certain		Male	11	53	sv-PPA	61	Negative		Not lv-PPA; Not nfv-PPA	**sv-PPA**	0.04	0.02	0.94
sv-PPA case 5	Certain		Male	11.5	69	sv-PPA	46	Negative	FTLD-TDP Type C	Uncertain	Not sv-PPA	0.25	0.06	0.69
sv-PPA case 6	Certain		Female	9.5	63	sv-PPA	64	Negative		Not lv-PPA; Not nfv-PPA	**sv-PPA**	0.10	0.07	0.83
sv-PPA case 7	Certain		Female	12.5	65	Not available	60	Negative		Not lv-PPA; Not nfv-PPA	**sv-PPA**	0.04	0.03	0.93
sv-PPA case 8	Certain		Female	9	58	Not available	44	Negative		Not lv-PPA; Not nfv-PPA	**sv-PPA**	0.19	0.01	0.79
sv-PPA (right) case 9	Certain		Female	6	73	sv-PPA	74	Negative		Uncertain	Not sv-PPA	0.09	0.12	0.71
sv-PPA case 10	Uncertain		Female	12	81	Not available	58	N/A	FTLD-TDP Type C	Not lv-PPA; Not nfv-PPA	**sv-PPA**	0.06	0.04	0.89
sv-PPA case 11	Uncertain	AD	Male	9	71	sv-PPA	48	Negative		Not lv-PPA; Not nfv-PPA	**sv-PPA**	0.11	0.06	0.83

sv-PPA case 12	Uncertain	lv-PPA	Male	18	75	lv-PPA	84	Positive	Uncertain	Not sv-PPA	0.10	0.12	0.54
sv-PPA case 13	Certain		Male	11	57	sv-PPA	62	Positive	Not lv-PPA; Not nfv-PPA	**sv-PPA**	0.06	0.02	0.91

Note, all PPA patients completed the Addenbrooke's Cognitive Examination-Revised (ACE-R). Details on how ACE-R performance scores were converted to equivalent ACE-III scores are reported in Supplementary Table 7. Patients shaded in grey were deemed atypical due to either the neurologist's clinical opinion or atypical findings on PiB-PET or brain autopsy. ^ Patient 12 was included in the initial ACE-III evaluation and modelled data (i.e., Phase 1) but was also included in Phase 2 based on the recent availability of autopsy findings. Abbreviations: * = co-occurring Lewy body pathology; ACE-III = Addenbrooke's Cognitive Examination-Third edition; ACE-R = Addenbrooke's Cognitive Examination-Revised; AD = Alzheimer's disease; CBS = corticobasal syndrome; Dx according to the neurologist = the most up-to-date clinical diagnosis according to the neurologist based on the patients' clinical history, brain imaging, and most recent clinical and neuropsychological assessment; Education = total years (y) of formal education; FTLD = frontotemporal lobar degeneration; lv-PPA = logopenic variant of primary progressive aphasia; nfv-PPA = non-fluent variant of primary progressive aphasia; PALS = progressive aphasia language scale; PiB-PET = [11C] Pittsburgh compound B positron emission tomography; PSP = progressive supranuclear palsy; sv-PPA = semantic variant of primary progressive aphasia; tau^1 = corticobasal degeneration type of tau pathology; tau^2= globular glial tauopathy ; tau^3 = progressive supranuclear palsy type of tau pathology; tau^4 = Pick's type of tau pathology; TDP-43 = Transactive response DNA binding protein-43.

Supplementary Table 7. How ACE-R performance scores in the PiB-PET or pathology confirmed PPA patients were converted to equivalent ACE-III performance scores (Phase 2)

ACE-III Item	How ACE-R performance scores were converted to ACE-III scores
Attention_Time	Same as ACE-III (no conversion)
Attention_Geography	Same as ACE-III (no conversion)
Attention_3_Word_Registration	Same as ACE-III (no conversion)
Attention_Serial_7s	Same as ACE-III (no conversion)
Memory_3_Word_Recall	Same as ACE-III (no conversion)
Fluency_Letter_Score	Same as ACE-III (no conversion)
Fluency_Animal_Score	Same as ACE-III (no conversion)
Memory_Learning_Address	Same as ACE-III (no conversion)
Memory_Retrograde	Same as ACE-III (no conversion)
Language_3_Commands	Performance scores on the ACE-R three-stage command were used for the ACE-III three single-step item.
Language_Writing	The ACE-R writing item is out of /1 but the ACE-III writing item is out of /2. ACE-R performance scores were converted to equivalent ACE-III scores: If the patient scored 1/1 on the ACE-R, then their performance was converted to 2/2. If the patient scored 0/1 on the ACE-R, then their score on the ACE-III writing item remained as 0/2.
Language_Repetition_Single_Words	Same as ACE-III (no conversion)
Language_Repetition_Glitters	Performance scores on the ACE-R sentence item 'Above beyond and below' were used for the ACE-III sentence item 'All that glitters is not gold’
Language_Repetition_A_Stitch	Performance scores on the ACE-R sentence item 'No ifs and or buts' were used for the ACE-III sentence item 'A stitch in time saves nine'
Language_Confrontation_Naming	Similar to ACE-III* (no conversion)
Language_Semantics	Same as ACE-III (no conversion)
Language_Reading	Same as ACE-III (no conversion)

Visuospatial_Loops	Performance scores on the ACE-R intersecting pentagons item were used for the ACE-III intersecting infinity loops item
Visuospatial_Cube	Same as ACE-III (no conversion)
Visuospatial_Clock	Same as ACE-III (no conversion)
Visuospatial_Dots	Same as ACE-III (no conversion)
Visuospatial_Letters	Same as ACE-III (no conversion)
Memory_Recall_Address	Same as ACE-III (no conversion)
Memory_Recognition_Address	Same as ACE-III (no conversion)

Notes: * The first two naming items on the ACE-R ("pencil" and "watch") were replaced/superseded in the ACE-III with other highly familiar objects ("spoon" and "book"). All other naming items in the ACE-R and ACE-III are the same.

Supplementary Table 8. Applying the PPA ACE-III diagnostic calculator in a sample of Alzheimer's disease patients (Phase 2)

Patient	Sex	Disease duration (years)	ACE-III Total	PiB-PET result	ACE-III PPA diagnostic calculator				
					lv-PPA vs. nfv-PPA calculator prediction	sv-PPA vs. non sv-PPA calculator prediction	lv-PPA probability	nfv-PPA probability	sv-PPA probability
AD case 1	Male	7.82	74	Positive	Uncertain	Not sv-PPA	0.6	0.21	0.07
AD case 2	Male	7.03	45	Positive	**lv-PPA**	Not sv-PPA	0.75	0.14	0.11
AD case 3	Male	5.57	67	N/A	Not lv-PPA; Not nfv-PPA	**sv-PPA**	0.18	0.03	0.75
AD case 4	Male	4.30	51	N/A	**lv-PPA**	Not sv-PPA	0.92	0.03	0.04
AD case 5	Male	10.17	73	N/A	Uncertain	Not sv-PPA	0.42	0.08	0.36
AD case 6	Male	13.17	65	N/A	Uncertain	Not sv-PPA	0.4	0.06	0.48
AD case 7	Male	6.23	62	Positive	**lv-PPA**	Not sv-PPA	0.8	0.11	0.07
AD case 8	Female	4.97	51	N/A	**lv-PPA**	Not sv-PPA	0.97	0.01	0.01
AD case 9	Male	6.96	74	N/A	Uncertain	Not sv-PPA	0.36	0.12	0.36
AD case 10	Male	3.94	72	N/A	Uncertain	Not sv-PPA	0.48	0.05	0.36
AD case 11	Male	4.06	71	N/A	Uncertain	Not sv-PPA	0.2	0.66	0.11
AD case 12	Female	2.06	58	Positive	**lv-PPA**	Not sv-PPA	0.73	0.16	0.09
AD case 13	Female	3.05	85	N/A	Uncertain	Not sv-PPA	0.38	0.15	0.11
AD case 14	Male	3.23	56	N/A	**lv-PPA**	Not sv-PPA	0.94	0.03	0.03
AD case 15	Female	2.25	70	N/A	Uncertain	Not sv-PPA	0.56	0.1	0.27
AD case 16	Female	2.61	78	N/A	Uncertain	Not sv-PPA	0.29	0.05	0.42
AD case 17	Male	2.79	59	N/A	Uncertain	Not sv-PPA	0.41	0.16	0.42
AD case 18	Male	1.79	57	N/A	Uncertain	Not sv-PPA	0.67	0.24	0.09
AD case 19	Male	2.72	40	N/A	**lv-PPA**	Not sv-PPA	0.76	0.17	0.06
AD case 20	Male	4.19	82	Positive	Uncertain	Not sv-PPA	0.27	0.16	0.29
AD case 21	Female	1.08	62	N/A	Uncertain	Not sv-PPA	0.23	0.1	0.64
AD case 22	Male	2.17	49	N/A	Uncertain	Not sv-PPA	0.61	0.07	0.31
AD case 23	Female	1.26	65	N/A	Uncertain	Not sv-PPA	0.56	0.08	0.33
AD case 24	Female	2.52	73	N/A	Uncertain	Not sv-PPA	0.45	0.04	0.38
AD case 25	Female	1.19	71	N/A	**lv-PPA**	Not sv-PPA	0.83	0.06	0.05
AD case 26	Female	2.87	62	N/A	Uncertain	Not sv-PPA	0.44	0.26	0.29

AD case 27	Female	2.52	71	N/A	Uncertain	Not sv-PPA	0.54	0.26	0.13
AD case 28	Female	6.40	69	N/A	**lv-PPA**	Not sv-PPA	0.78	0.07	0.06
AD case 29	Male	6.05	60	N/A	Uncertain	Not sv-PPA	0.2	0.31	0.49
AD case 30	Female	4.47	67	N/A	Uncertain	Not sv-PPA	0.58	0.16	0.21
AD case 31	Female	4.55	48	N/A	**lv-PPA**	Not sv-PPA	0.78	0.22	0.01
AD case 32	Male	1.93	68	N/A	Uncertain	Not sv-PPA	0.65	0.03	0.24
AD case 33	Male	1.37	69	N/A	**lv-PPA**	Not sv-PPA	0.7	0.08	0.16
AD case 34	Male	3.74	55	N/A	Uncertain	Not sv-PPA	0.48	0.38	0.14
AD case 35	Male	2.35	59	N/A	Uncertain	Not sv-PPA	0.63	0.25	0.1
AD case 36	Male	3.29	49	N/A	**lv-PPA**	Not sv-PPA	0.96	0.03	0.01
AD case 37	Male	5.16	81	N/A	Uncertain	Not sv-PPA	0.48	0.12	0.12
AD case 38	Female	3.30	73	N/A	Uncertain	Not sv-PPA	0.21	0.09	0.67
AD case 39	Female	7.74	58	N/A	Not lv-PPA; Not nfv-PPA	**sv-PPA**	0.09	0.04	0.86
AD case 40	Male	7.45	58	N/A	Uncertain	Not sv-PPA	0.57	0.3	0.12
AD case 41	Male	9.59	52	N/A	Uncertain	Not sv-PPA	0.68	0.14	0.17
AD case 42	Female	2.60	73	N/A	Uncertain	Not sv-PPA	0.5	0.08	0.3
AD case 43	Male	4.13	60	N/A	Uncertain	Not sv-PPA	0.68	0.15	0.15
AD case 44	Male	2.09	57	N/A	Uncertain	Not sv-PPA	0.34	0.11	0.54
AD case 45	Male	3.36	78	N/A	Uncertain	Not sv-PPA	0.4	0.04	0.39
AD case 46	Female	3.33	47	N/A	**lv-PPA**	Not sv-PPA	0.88	0.1	0.02
AD case 47	Male	4.09	44	N/A	**lv-PPA**	Not sv-PPA	0.82	0.13	0.05
AD case 48	Female	1.31	79	N/A	Uncertain	Not sv-PPA	0.35	0.08	0.29
AD case 49	Male	5.18	65	N/A	**lv-PPA**	Not sv-PPA	0.74	0.11	0.11
AD case 50	Male	4.94	81	N/A	Uncertain	Not sv-PPA	0.31	0.16	0.17
AD case 51	Male	3.80	73	N/A	Uncertain	Not sv-PPA	0.34	0.42	0.14
AD case 52	Female	4.79	64	N/A	**lv-PPA**	Not sv-PPA	0.83	0.06	0.08
AD case 53	Female	4.39	46	N/A	**lv-PPA**	Not sv-PPA	0.86	0.06	0.08
AD case 54	Male	3.00	70	N/A	Uncertain	Not sv-PPA	0.6	0.08	0.26
AD case 55	Female	3.21	65	N/A	Uncertain	Not sv-PPA	0.27	0.14	0.53
AD case 56	Male	4.60	56	N/A	Uncertain	Not sv-PPA	0.56	0.32	0.12
AD case 57	Male	4.95	79	N/A	Uncertain	Not sv-PPA	0.38	0.05	0.36

AD case 58	Female	1.67	59	N/A	Uncertain	Not sv-PPA	0.62	0.08	0.29
AD case 59	Male	1.20	90	N/A	Uncertain	Not sv-PPA	0.18	0.06	0.1
AD case 60	Male	3.35	82	N/A	Uncertain	Not sv-PPA	0.32	0.09	0.19
AD case 61	Female	5.42	71	N/A	Uncertain	Not sv-PPA	0.36	0.15	0.36
AD case 62	Female	3.54	83	N/A	Uncertain	Not sv-PPA	0.31	0.07	0.12
AD case 63	Female	2.55	54	N/A	**nfv-PPA**	Not sv-PPA	0.17	0.72	0.1
AD case 64	Female	2.87	43	N/A	**lv-PPA**	Not sv-PPA	0.91	0.04	0.05
AD case 65	Male	4.88	74	N/A	Uncertain	Not sv-PPA	0.44	0.39	0.08
AD case 66	Male	2.96	71	N/A	Uncertain	Not sv-PPA	0.33	0.03	0.57
AD case 67	Male	1.54	54	N/A	Uncertain	Not sv-PPA	0.52	0.41	0.05
AD case 68	Male	4.13	52	N/A	**lv-PPA**	Not sv-PPA	0.91	0.03	0.06

Notes: All Alzheimer's disease patients completed the Addenbrooke's Cognitive Examination-Third edition. Abbreviations: ACE-III = Addenbrooke's Cognitive Examination-Third edition; AD = Alzheimer's disease; Disease duration = time (years) since the onset of symptoms as described by the caregiver; lv-PPA = logopenic variant of primary progressive aphasia; nfv-PPA = non-fluent variant of primary progressive aphasia; PiB = [11C] Pittsburgh Compound B; sv-PPA = semantic variant of primary progressive aphasia

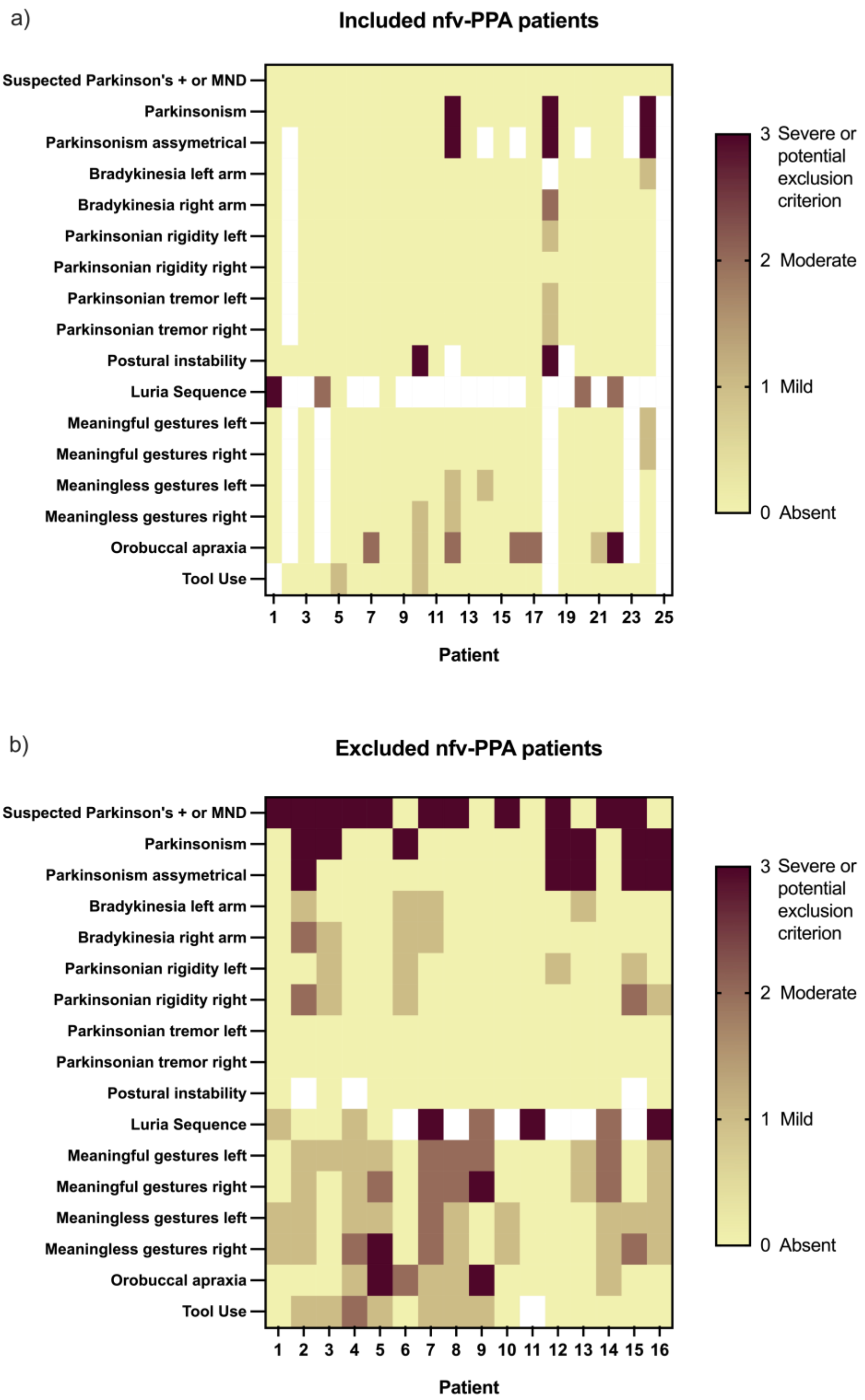

Supplementary Figure 1. Phase 1 nfv-PPA patients a) included or b) excluded based on the presence or absence of parkinsonian and/or motor neuron features at baseline assessment (i.e., at the time of the first ACE-III assessment).

Notes: White spaces indicate unavailable data. 'Suspected Parkinson's + or MND' = suspected Parkinson's plus or motor neuron disease according to the neurologist's (R.M.A., N.J.C, E.D., Y.T.H., J.R.H., J.R.B.) clinical notes. The parkinsonism, parkinsonism asymmetrical and postural instability

items were recorded as a ‘yes’ or ‘no’ by the neurologist. ‘Yes’ responses for these items were maroon colour coded (i.e., ‘potential exclusion criteria). All other items were rated as absent (0), mild (1), moderate (2), or severe (3).

In Figure 1a), patients 12, 18 and 24 had mild parkinsonism. In all cases, these features were asymmetrical (e.g., left side < right side). Further, patients 10 and 18 had postural instability. It was decided, however, that these features were not in keeping with a clear parkinsonian syndrome. Patient 25 did not undergo formal assessment for parkinsonism at our research clinic but was informally assessed by an experienced neurologist (R.M.A) at another clinic. In Figure 1b), 11/16 patients (i.e., patients 1-5, 7-8, 10, 12, 14-15) had features in keeping with a concomitant parkinsonian or motor neuron syndrome, and a further 5 patients (patients 6, 9, 11, 13, 16) had suspected (but unclear) parkinsonian or motor neuron features. Admittedly, while patient 11 went on to develop corticobasal syndrome at a follow up assessment, their parkinsonian features were very mild at baseline assessment and so should have been included in the Phase 1 data analysis. Fifteen out of the 16 patients in Figure 1b) went on to develop clear Parkinson’s plus (corticobasal syndrome: patients 1-8, 12, 14 and 15; progressive supranuclear palsy: patient 16) or motor neuron disease syndromes (patients 9, 10, 11) within 1-2 years post baseline assessment.

Supplementary Material Appendix 1

Instructions of the Progressive Aphasia Language Scale (PALS)

I. Spontaneous speech

Based upon 10 minutes of informal conversation on topics, such as previous occupation, hobbies, travel, and family, during which the examiner encourages free utterances and interrupts as little as possible.

1. Motor speech disorders. Features to observe:

   A. Distortions, articulatory groping and laboured speech.
   B. Changes in prosody, rhythm, intonation, and stress.
   C. Intelligibility of words.

2. Agrammatism. Features to observe:

   A. Omission of grammar structures, such as prepositions, articles, pronouns, and auxiliary verbs. Simplification and telegraphic speech.
   B. Incorrect inflection of verbs and other grammatical structures. Wrong usage of pronouns and arrangement of words.

II. Tasks

3. Naming: The subject is asked to name 10 animals (and then 10 tools).

   A. Animal models: Tiger, elephant, crocodile, kangaroo, giraffe, zebra, goat, sheep, horse, and donkey.
   B. Household objects: scissors, tongs, spoon, whisk, toothbrush, nutcracker, spanner, potato masher, grater, and can-opener.

The examiner notes the types of errors –omission, semantic associative, or phonological. If errors are made in the naming, comprehension is tested by asking the subject to point to the 'crocodile', 'giraffe', and so forth. Then, knowledge is tested in all subjects by asking them to repeat the word (e.g., '*domesticated'*) and to point to the relevant animals, etc. (see 5A)

4. Single-word repetition (and definitions, see task 5B). The subject is asked to repeat (and then to define) words of increasing length and phonological complexity:

   - '*Banana'*
   - *'Potato'*
   - *'Methodist'*
   - *'Artillery'*
   - *'Perimeter'*
   - *'Caterpillar'*
   - *'Catastrophe'*
   - *'Chrysanthemum'*

As with naming, the type of errors should be noted.

5. Single-word comprehension. This component is assessed simultaneously with the previous tasks:
    A. Selection of objects shown in the task 3: The subject is asked to select the animals according to the specified category; e.g., which one of these animals are '*edible*', '*ferocious*', '*domesticated*', '*indigenous*', '*herbivorous*'.
    B. Definition of words given in the task 4: After repeating the word '*catastrophe*', the subject is asked to define it: What sort of thing is '*catastrophe*'? The examiner can classify the subject's knowledge by probing or asking yes/no questions.

Word comprehension is judged, therefore, on three factors: pointing to the correct items if they make naming errors (e.g., point to the '*crocodile'*); comprehension of words like '*domesticated*', '*edible*'; and definition of the words in the repeat and define task.

6. Sentence repetition. Subjects are asked to simply repeat a series of phrases or sentence:
    - *'Not ifs ands or buts'*
    - *'The Chinese fan contained a rare emerald'*
    - *'Six small boys built a sizeable snowman'*
    - '*The lady delivered some delicious gingerbread'*

Patients may omit words or produce errors as in naming and word repetition.

7. Sentence comprehension. The examiner places three common objects (e.g., pen, toothbrush, and spoon).

    A. Simple instructions: Direct two-step commands, such as '*touch the pen then the toothbrush'; 'pick up the pen and then the spoon';* etc. Typically, three such commands are given. If correct pass part B.
    B. Complex instructions: These include indirect instructions (e.g., *'pass me the pen after touching the toothbrush'; 'pick up the spoon after touching the pen'*) and instructions with embedded clauses (e.g., '*give me all the items that you find in the kitchen'*).

REFERENCES

1. Gorno-Tempini ML, Hillis AE, Weintraub S, et al. Classification of primary progressive aphasia and its variants. *Neurology*. Mar 15 2011;76(11):1006-14. doi:WNL.0b013e31821103e6 [pii] 10.1212/WNL.0b013e31821103e6

2. Wechsler D. *Wechsler Memory Scale: Administration and Scoring Manual*. 3rd ed. The Psychological Corporation.; 1997.

3. Rey A. L'examen psychologique dans les cas d'encéphalopathie traumatique. *Archives de psychologie*. 1941;28:286-340.

4. Savage S, Hsieh S, Leslie F, Foxe D, Piguet O, Hodges JR. Distinguishing subtypes in primary progressive aphasia: application of the Sydney language battery. Research Support, Non-U.S. Gov't. *Dementia and geriatric cognitive disorders*. 2013;35(3-4):208-18. doi:10.1159/000346389

5. Strauss E, Sherman EMS, Spreen O. *A compendium of neuropsychological tests: Administration, norms, and commentary, 3rd ed*. A compendium of neuropsychological tests: Administration, norms, and commentary, 3rd ed. Oxford University Press; 2006:xvii, 1216-xvii, 1216.

6. Benton AL, Hamsher, K. deS., & Sivian, A.B. *Multilingual Aphasia Examination (3rd ed.)*. AJA Associates, Inc.; 1994.

7. Reitan RM. The relation of the trail making test to organic brain damage. *Journal of Consulting Psychology*. Oct 1955;19(5):393-4.

8. Miller LA, Hsieh S, Lah S, Savage S, Hodges JR, Piguet O. One size does not fit all: face emotion processing impairments in semantic dementia, behavioural-variant frontotemporal dementia and Alzheimer's disease are mediated by distinct cognitive deficits. *Behavioural neurology*. 2012;25(1):53-60.

9. Kumfor F, Sapey-Triomphe L-A, Leyton CE, Burrell JR, Hodges JR, Piguet O. Degradation of emotion processing ability in corticobasal syndrome and Alzheimer's disease. *Brain*. 2014;137(11):3061-3072.
